# Magnetically Driven Micro and Nanorobots

**DOI:** 10.1021/acs.chemrev.0c01234

**Published:** 2021-03-31

**Authors:** Huaijuan Zhou, Carmen C. Mayorga-Martinez, Salvador Pané, Li Zhang, Martin Pumera

**Affiliations:** † Center for Advanced Functional Nanorobots, Department of Inorganic Chemistry, University of Chemistry and Technology Prague, Technicka 5, 166 28 Prague 6, Czech Republic; ‡ Multi-Scale Robotics Lab (MSRL), Institute of Robotics and Intelligent Systems (IRIS), ETH Zurich, Tannenstrasse 3, 8092 Zurich, Switzerland; § Department of Mechanical and Automation Engineering, The Chinese University of Hong Kong, Hong Kong 999077, China; ⊥ Department of Medical Research, China Medical University Hospital, China Medical University, No. 91 Hsueh-Shih Road, Taichung 40402, Taiwan; ¶ Department of Chemistry and Biochemistry, Mendel University in Brno, Zemedelska 1, CZ-613 00 Brno, Czech Republic; # Department of Chemical and Biomolecular Engineering, Yonsei University, 50 Yonsei-ro, Seodaemun-gu, Seoul 03722, Korea; ∥ Future Energy and Innovation Laboratory, Central European Institute of Technology, Brno University of Technology, Purkyňova 656/123, Brno CZ-612 00, Czech Republic

## Abstract

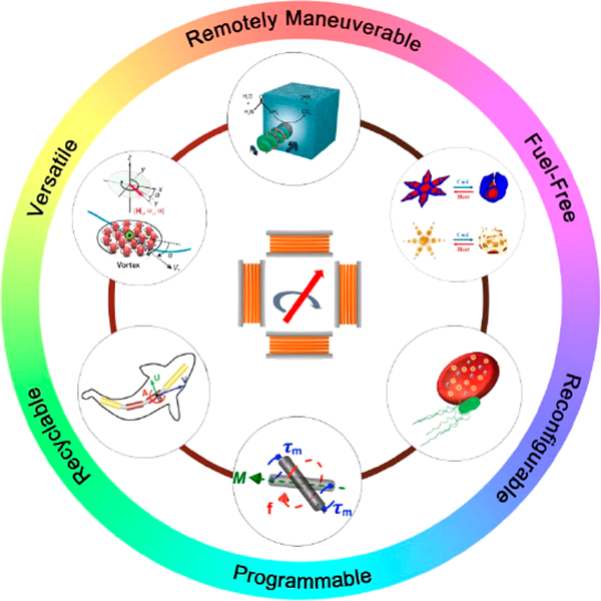

Manipulation and navigation of micro and nanoswimmers in different fluid environments can be achieved by chemicals, external fields, or even motile cells. Many researchers have selected magnetic fields as the active external actuation source based on the advantageous features of this actuation strategy such as remote and spatiotemporal control, fuel-free, high degree of reconfigurability, programmability, recyclability, and versatility. This review introduces fundamental concepts and advantages of magnetic micro/nanorobots (termed here as “MagRobots”) as well as basic knowledge of magnetic fields and magnetic materials, setups for magnetic manipulation, magnetic field configurations, and symmetry-breaking strategies for effective movement. These concepts are discussed to describe the interactions between micro/nanorobots and magnetic fields. Actuation mechanisms of flagella-inspired MagRobots (i.e., corkscrew-like motion and traveling-wave locomotion/ciliary stroke motion) and surface walkers (i.e., surface-assisted motion), applications of magnetic fields in other propulsion approaches, and magnetic stimulation of micro/nanorobots beyond motion are provided followed by fabrication techniques for (quasi-)spherical, helical, flexible, wire-like, and biohybrid MagRobots. Applications of MagRobots in targeted drug/gene delivery, cell manipulation, minimally invasive surgery, biopsy, biofilm disruption/eradication, imaging-guided delivery/therapy/surgery, pollution removal for environmental remediation, and (bio)sensing are also reviewed. Finally, current challenges and future perspectives for the development of magnetically powered miniaturized motors are discussed.

## 1 Introduction

Many species in nature, such as magnetotactic bacteria, birds, bats, butterflies, lobsters, and salmon, can fly or swim over a long distance by perceiving navigation cues from geomagnetic fields. Some species (e.g., *Amitermes meridionalis*) even have the ability to (re)orient their bodies or nests according to geomagnetic information. Similarly, the locomotion of nanoscale and microscale objects in a predefined path by the navigation of magnetic fields,^1−4^ which are mainly generated by moving charges (i.e., electric currents) and magnetic materials (such as permanent magnets), has drawn extensive attention owing to their tremendous potential for applications in biomedicine and environmental remediation. Such miniaturized objects are normally termed as “magnetically driven micro/nanorobots” (called “MagRobots” for short in this review), which is an important branch of micro and nanorobots.

Micro/nanorobots are locomotive artificial machines with size in the micro or nanoscale and rationally designed to execute tasks on command via self-propulsion or an externally controlled propulsion mechanism. Ideally, micro/nanorobots should have the ability to undertake tasks via encapsulation/functionalization with diagnostic or therapeutic agents, decoration with functional materials, or being fabricated into special micro/nano architectures; “delivery tasks” by moving toward targeted sites in a user-defined path or a theoretically and experimental optimized path; “execute tasks”, for example, killing diseased cells/tissues, removing environmental pollutants as required; and “exit tasks” after the task accomplishment via recycling or *in situ* degradation. During task implementation, locomotion behavior is of great importance for micro and nanorobots. The migration of micro and nanorobots can be powered by multiple strategies including chemical catalysis (e.g., O_2_ or H_2_ generation) or chemical gradients,^5−11^ external energy sources (e.g., magnetic field,^12−14^ light,^15−21^ acoustic wave,^22−25^ or electrical field^26−28^), and even motile cells (e.g., sperm cell, bacterial cell).^29−37^ According to the power source, micro/nanorobots can be classified as chemically driven (or fuel-driven), magnetically driven, light-driven, ultrasound-driven, electrically driven. The word “driven” can be replaced by “powered”, “actuated”, or “propelled”. According to their functionalities, micro/nanorobots can be named as micro/nanogrippers,^38−40^ micro/nanodrillers,^41^ micro/nanocleaners,^42,43^ micro/nanoscavengers,^44^ etc. Readers can refer to our latest review^45^ to obtain a more detailed classification of micro/nanorobots based on geometric shapes, motion modes, and functionalities.

Chemically propelled micro/nanorobots are faster than those with other propulsion methods, but their locomotion lacks directionality. Moreover, they require toxic fuels such as H_2_O_2_, N_2_H_4_, HCl, urea, and NaBH_4_.^46,47^ In comparison, those micro/nanorobots powered by external physical fields (such as magnetic, ultrasound, light, and electric fields) do not need toxic chemical fuels for propulsion, but their motion is relatively slow.^48−52^ Light-propelled micro/nanorobots can move in water; however, depending on their composition, they need H_2_O_2_ and a high-intensity light source, which could compromise their biocompatibility. On the other hand, micro/nanomotors propelled by ultrasound are biocompatible but lack directionality control, making it difficult for them to perform specific tasks. Finally, micro/nanomotors propelled by electric field are very promising for fuel-free locomotion; however, its biological application is still limited and not yet fully demonstrated. Magnetically driven micro/nanomotors address most disadvantages presented by others propulsion principles and, until now, have been the more explored and used in many biomedical applications as well as for environmental control and remediation. Furthermore, magnetic medical microrobots can be driven by magnetic resonance imaging (MRI) systems, thus utilizing existing clinical MRI equipment for dual purposes, namely the imaging and tracking of microrobots, and their propulsion and motion control.^53,54^ Likewise, clinical ultrasonography systems hold great potential to actuate ultrasonically driven microrobots.^45^

In addition, among all the actuation strategies, the utilization of a magnetic field for manipulating miniaturized robots has unparalleled advantages, which are summarized as follows. (i) Remote maneuverability: magnetic fields provide a noninvasive way to manipulate matter owing to the inherent contactless characteristics of magnetic forces. Such a wireless actuation method allows for micro and nano agents to move in an untethered manner while keeping their local chemical environment intact. (ii) Fuel-Free: using a magnetic field for propulsion is a clean process that does not consume liquid fuel (unlike for chemically and photochemically propelled swimmers). This feature eliminates the harmful effects of toxic chemicals (e.g., hydrogen peroxide) on cells and tissues during their biological application processes. In addition, magnetic fields exhibit insignificant dependence on features and properties of surrounding environments and cause negligible damage to cells at low frequencies. (iii) Reconfigurability and programmability of magnetic materials: reconfigurability refers to the rearrangement of the swimmer’s features such as the morphology, locomotion mode, or other motion parameters upon the application of magnetic fields or other external stimuli. Examples of reconfigurable structures are magnetically driven particulate swarms,^55−57^ stimuli-responsive magnetic materials (i.e., ferromagnetic shape-memory alloys), or composite structures (i.e., smart magneto-polymer composites^58,59^ or complex origami-like architectures^60^). This type of structure can readily change its shape by changing the conditions of the applied magnetic fields (i.e., frequency or magnitude). Programmability refers to the ability to manipulate the components of the MagRobots in terms of their shape, magnetic shape, magnetic anisotropy,^61^ and crystalline anisotropy to achieve a specific motion mode, position, or orientation when magnetic fields are applied.^62,63^ For example, the orientation of a magnetic composite-based structure can be programmed by suitably aligning the particles within the composite matrix.^60^ Specific shape-morphing small-scale systems can also be designed to exhibit both reconfigurability and programmability.^64^ (iv) Recyclability of magnetic materials: after micro/nanorobots have completed their tasks, the separation and recycling of introduced foreign matter from water, biological fluids, or even tissues might be necessary in terms of biosafety and biocompatibility. Magnetic nano/microrobots, as they are composed of magnetic building blocks (i.e., coating, segment, particulates), allow for a feasible and convenient magnetically assisted retrieval and recycling process. (v) Versatility: by combining a magnetic field with other actuation sources, the transport and delivery of functional cargos (e.g., drugs or a single cell at the nanosize level) can be achieved with high maneuverability and sensitivity.^65^ Currently, various hybrid power sources, such as magneto-acoustic,^22,23,66^ magneto-optical,^67^ and magneto-chemotaxis,^68^ have been reported, which provide dual propulsion modes in response to multiple stimuli.

Molecular machines are molecular components capable of implementing mechanical locomotion (as output) in response to particular external stimuli (as input).^69−72^ Stimuli can be various energy inputs such as chemical energy, electric energy, light, photochemical, electrochemical energy, or pH gradient.^73−77^ Although molecular machines can perform very complicated functions, most functions are limited to conformational movements.^78−82^ In terms of practical uses, particularly for biomedical applications, the operator’s real-time imaging and tracking of the tiny robots are required when they are carrying out specific tasks inside the human body.^10,83^ This requirement may limit the applicability of molecular machines due to their nanoscale (<10 nm) size being too small to be readily visualized using traditional imaging techniques. By contrast, larger micro- and nanorobots can provide greater feasibility for bioimaging for the applications in medical fields.^53,84−86^ To this end, swarms of micro/nanorobots can also be used for their imaging and positioning abilities.^87−89^

Recent reviews about micro and nanorobots that focus on fabrication techniques,^51^ geometric shapes (e.g., active particles,^90^ Janus,^91^ tubular,^92^ hybrid actuators^81,93^), actuation sources (e.g., light,^48,49^ magnetic field^94^), propulsion mechanisms,^82^ and potential applications (e.g., cancer therapy^95^) provide us with a basic understanding and up-to-date developments in this multidisciplinary and interdisciplinary area. A comprehensive understanding of how tiny machines behave under magnetic fields will inspire and trigger interdisciplinary and cross-disciplinary scientific and technological innovation for multiple applications. The goal of this review is to provide a general view of the locomotion behaviors of nano and microscale motors under the manipulation of a magnetic field and guidance for their rational design by describing the interaction of MagRobots and magnetic fields as well as actuation and movement mechanisms, and reporting state-of-the-art fabrication techniques. After demonstrating current applications in biological and environmental fields, a further outlook of this new and exciting field is presented.

## 2 Interations between Micro/Nanorobots and Magnetic Fields

### 2.1 Magnetic Fields and Magnetic Materials

Magnetic fields, as vector-valued functions of the position, originate from the movement of electric charge. Magnetic fields can be generated by two distinct sources: freely moving electric currents and magnetic materials. Typically, the former source is generated by the coil of an electromagnet that is externally controllable. The setups of a triaxial orthorhombic Helmholtz coil and eight electromagnetic coils (e.g., MiniMag, OctoMag) are representative and widely employed to generate magnetic fields for driving and steering MagRobots (see Section 2.2). The latter source is generated from the intrinsic magnetization of magnetic materials, specifically permanent ferromagnets, which can retain a large remnant magnetization. To manipulate micro- and nanomachines by magnetic fields, a conventional strategy consists of incorporating magnetic components into nano/microstructures. Magnetic materials can be classified as a function of the magnetic susceptibility (*x*_*m*_), a parameter that reflects how easy a magnetic material is magnetized. As such, magnetic materials are categorized as ferromagnetic (and ferrimagnetic) materials (*x*_*m*_ ≫ 0), paramagnetic materials (*x*_*m*_ > 0), and diamagnetic materials (*x*_*m*_ < 0). Paramagnets and diamagnets are weakly attracted or repelled, respectively, to magnetic fields. Additionally, they cannot retain any magnetization once the magnetic field is removed. Ferro- and ferrimagnets are all strongly attracted to magnetic fields. Specifically, ferro- and ferrimagnets can retain magnetization, (i.e., exhibit remnant magnetization or remanence) after being subjected to a magnetic field. Usually, high remanence is a feature of hard-ferromagnetic materials, otherwise known as permanent magnets. Soft-ferromagnets, in contrast, exhibit low remanence. Both soft- and hard-magnets exhibit a hysteretic behavior, which means that to demagnetize these materials, a coercive magnetic field is necessary. This coercivity is large for hard-magnets and small for soft-magnets. Superparamagnets are a special class of materials in which features of both ferromagnets and paramagnets converge such as high susceptibility, no remanence, and no coercivity. While a few examples exist of micro/nanorobots constructed of paramagnets and diamagnets,^96,97^ the majority of magnetic small-scale robots have been made of ferromagnetic, ferrimagnetic, and superparamagnetic compounds. For extended details on types of magnetic materials, we suggest the reader to review the hereby indicated references.^98−100^

When placing a magnetic small-scale robot with a volume *v* in an external magnetic field ***B***, the device will display a magnetization ***M***. If the device is subject to a magnetic field gradient Δ*B*, it will experience an attractive force (or repulsive if it is a diamagnet) as expressed in eq 1. If the device is subjected to a magnetic field, to minimize its energy, it will experience a torque as expressed in eq 2, which will cause the magnetic robot to orient in such a way that its easy magnetization axis is parallel to the direction of the applied magnetic field. The easy magnetization axis is usually governed by the shape (shape anisotropy) but can also be ruled by specific crystal orientations of the materials (crystalline anisotropy). Additionally, the easy magnetization axis can be programmed, for instance, by orienting magnetic nanostructures with a matrix of a composite component or by premagnetizing a material in a specific direction:
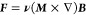
1
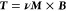
2

Both magnetic forces generated in gradient fields and magnetic torque induced by spatially homogeneous or heterogeneous dynamic fields can function as “fuel” to actuate microscopic and nanoscopic motors in various environments. In terms of magnetic torque, weak homogeneous rotating or oscillating fields (see Section 2.3), which display higher efficiency in transforming magnetic energy into kinetic energy, are highly preferable. Magnetic fields offer a maximum of six degrees of freedom (DoFs) (i.e., three translational DoFs and three rotational DoFs) for absolute spatial manipulation of micro/nanorobots, depending on the setup of electromagnetic actuation systems (see Section 2.2). For instance, the widely used uniform rotating magnetic field with triaxial Helmholtz coil can supply three rotational DoFs, while MiniMag and OctoMag have five DoFs: two rotational and three translational DoFs.

### 2.2 Magnetic Manipulation Systems

**Figure 1:**
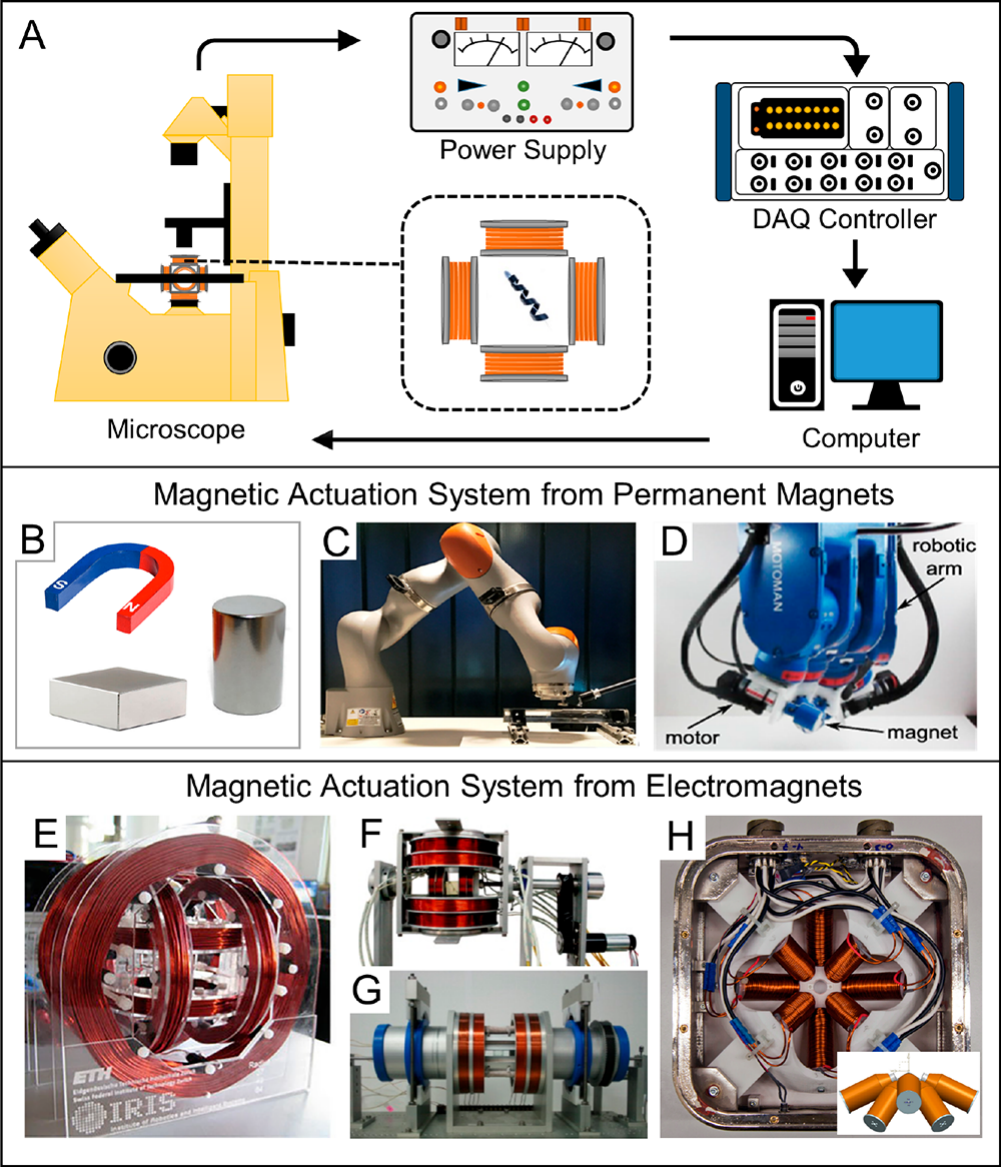
Experimental setup for magnetically driven micro/nanorobots and various magnetic actuation systems. (A) Diagram of the typical experimental workplace for actuating and visualizing MagRobots. (B) Magnetic actuation system consists of only a single permanent magnet. (C) Permanent magnet actuation system using cylindrical NdFeB permanent magnet fixed to its end-effector and a robotic arm. Reproduced with permission from ref (101). Copyright 2017 IEEE. (D) Rotating permanent magnet system consists of a magnet, a robotic arm, and a motor. Reproduced with permission from ref (102). Copyright 2013 IEEE. (E) Electromagnetic actuation system using triaxial circular Helmholtz coils. Reproduced with permission from ref (103). Copyright Springer Science + Business Media, LLC 2013. (F) Electromagnetic actuation system using a stationary Helmholtz–Maxwell coil and a rotational Helmholtz–Maxwell coil. Reproduced with permission from ref (104). Copyright 2009 Elsevier B.V. (G) Electromagnetic actuation system using multiply coils including a Helmholtz coil, Maxwell coil, uniform saddle coil, and gradient saddle coil. Reproduced with permission from ref (105). Copyright 2010 Elsevier B.V. (H) MiniMag electromagnetic system. Reproduced with permission from ref (106). Copyright 2014 Springer-Verlag GmbH Berlin Heidelberg.

A typical setup platform for monitoring and actuating magnetically driven micro- and nanorobots consists of a sample stage, an optical microscope (eventually, coupled with a high-resolution camera), a magnetic manipulation system, and a computer system with video capture and analysis (Figure 1A). The magnetic manipulation system consists of a set of either permanent magnets or electromagnets^107−110^ as the source of the magnetic field. Recent contributions^97,111,112^ provide a systematic review of configurations of magnetic manipulation systems that can be applied to magnetic small-scale robots with sizes ranging from nanometers to millimeters. In this review, we will only focus on the commonly used magnetic systems employed for the manipulation of nanoscale and microscale robots.

One of the main differences between systems using permanent magnets and electromagnets is the fact that the magnetic field from a permanent magnet is persistent and its magnitude cannot be quickly changed. The distribution and strength of a magnet’s field depend on its geometrical shape and size. For a magnetized object with a given geometry shape and magnetization, large magnets can project their field further into space. However, large magnets produce smaller magnetic forces as demonstrated in eq 1 because the change of field in space (i.e., spatial derivatives in the field) is less pronounced. By manually or automatically adjusting the position or orientation of a magnet, a translatory or rotational movement of MagRobots can be triggered. Direct utilization of portable magnet provides an easy-to-operate way to drive the motion of MagRobots by simply adjusting the position and orientation of a magnet (Figure 1B). Although many researchers have reported the locomotion of magnetic micro/nanorobots by using single permanent magnets, the experimental reproducibility and accuracy are challenging aspects because the movement of magnets largely depends on their operator. Given the drawbacks of manual handling, many automatically operable magnet systems have been designed by integrating a magnet with a commercial robotic arm such as the LBR Med robotic arm from KUKA Robotics Corporation (Figure 1C) and MH5 robotic arm from Yaskawa Motoman. Such an integrated system is more reliable and precise. Besides magnetic field gradients, magnetic torque can also be exerted on small-scale devices when the magnet rotates (Figure 1D), which allows for rotational actuation mechanisms.

In magnetic actuation systems based on electromagnets, magnetic fields are generated from flowing currents through coils. A typical electromagnet is formed by wrapping insulated copper wires around a ferromagnetic core, which can concentrate and amplify the magnetic field and field gradient. An ideal soft magnetic material is often used as the core in order to avoid effects of hysteresis. On-demand setting of current in each coil can result in the required configuration of magnetic fields, such as rotating field, oscillating field, alternating fields, and conical fields, which will be discussed in Section 2.3. Different arrangements of coils constitute specialized electromagnet systems such as the Helmholtz coil, the Maxwell coil, the saddle coil, and the double-saddle Golay coil (detailed information can be found in ref (113)). Helmholtz coil, containing two circular and coaxial coils with equal radius and same handedness of flowing current, is the first and most important arrangement. Because the field generated from the Helmholtz coil is near-uniform at the center of the coils, such a magnetic actuation system is appropriate for magnetic torque control.^114−117^ Arbitrary uniform magnetic fields in a 2D plane or 3D space can be generated by two pairs of Helmholtz coils or triaxial Helmholtz coils, respectively. Triaxial circular Helmholtz coils are the most commonly used for actuating magnetic small-scale robots (Figure 1E). The combination of Helmholtz coils with other types of coils can engender systems with multi-DOF capabilities. Maxwell coil is also composed of two circular coaxial coils with equal radius, but the current flowing through different coils coil has the opposite handedness. Maxwell coils can create uniform magnetic field gradients, saddle coils can generate a uniform field or a gradient field, and double-saddle Golay coils can produce a transverse gradient. A magnetic manipulation system with a stationary Helmholtz–Maxwell coil and a rotational Helmholtz–Maxwell coil has the capacity of 3D locomotion of a magnetic small-scale robot through the control of both magnetic forces and torques (Figure 1F).^104^ Its upgraded system using four different coil pairs (i.e., a Helmholtz coil, a Maxwell coil, a rotatory uniform saddle coil, and a rotatory gradient saddle coil) occupies a smaller volume and consumes less driving energy (Figure 1G).^105^ Given the practical clinical application of biomedical micro/nanorobots, saddle coil and Golay coil with tubular construction are preferable because they have high space efficiency and, hence, are capable of accommodating the human body. For example, a widely used magnetic resonance imaging (MRI) scanner in clinical practice incorporates a Maxwell coil and two orthogonal Golay coils.^118^

A drawback of magnetic actuation systems consisting of paired coils lies in their restrictions on the shape and size of the workspace. In contrast, electromagnetic systems using several nonorthogonally distributed electromagnets, usually made of columnar coils with soft-iron cores, can break this limitation by arranging the electromagnets so that their generated dipoles keep their respective axes pointing to a common point in the given workspace. The first example of such configuration was the OctoMag, an electromagnet comprising a total of eight electromagnets. OctoMag is a system capable of generating magnetic forces and torques in three dimensions and allows for a 5-DOF magnetic control (3-DOF position and 2-DOF orientation).^119^ OctMag is composed of four evenly distributed electromagnets in a plane with the orientation of 90° from a central axis and four evenly distributed electromagnets with the orientation of 45° from a central axis. MiniMag is the scaled-down compact version of the OctoMag (Figure 1H). Utilization of OctMag and MiniMag has been reported to remotely manipulate micro- and nanorobots for targeted drug delivery,^120^ minimally invasive ophthalmic surgery,^121^ and stem cell transplantation in a rat brain.^122^ Other configurations of electromagnets, such as square antiprism, cubic, open asymmetric, and so on, were summarized in a recent review.^113^

### 2.3 Actuation Configurations for MagRobots

**Figure 2:**
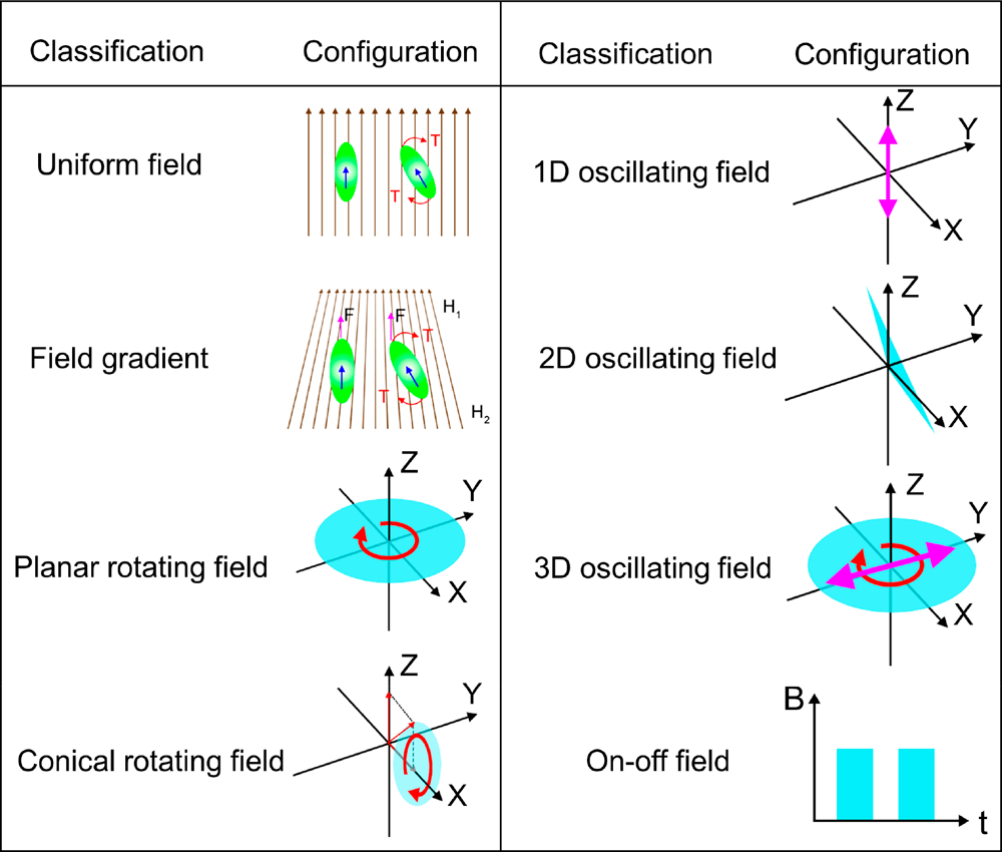
Classifications and configurations of magnetic fields in relation to the motion of MagRobots.

According to changes of the magnetic field vector with time, magnetic fields can be classified as static, dynamic (including a rotating magnetic field whose direction varies with time, an oscillating magnetic field whose strength varies with time), or on–off fields. Both static and dynamic magnetic fields can be homogeneous fields where the field vector modulus remains constant in space, or inhomogeneous magnetic fields where the field strength varies with position, that is, field gradient.^123^ Rotating magnetic fields are widely adopted to induce rotational motion. For some micro and nanomachines with specific shapes (e.g., helical structure), such temporal–periodic rotational motion can be converted into translational corkscrew motion (see Sections 3.1 and 4.2), which leads to a net spatial displacement. In contrast, oscillating magnetic fields can be utilized to activate traveling undulatory locomotion for some MagRobots such as those with soft tails (see Section 3.2) and those consisting of solid segments linked with soft hinges (see Section 4.4). Rotational magnetic fields can also induce thermophoretic motion for ferromagnetic materials by generating heat energy^124^ (see Section 3.4). Figure 2 summarizes different categories of magnetic fields and their corresponding field diagrams.^125^

### 2.4 Effective Movements in MagRobots: “Symmetry-Breaking Strategies”

To begin this section, we would like to briefly introduce the hydrodynamic laws to understand how small-scale robots swim in a fluid. The Navier–Stokes equation, arising from Newton’s second law, describes the motion of a Newtonian fluid as follows (eq 3):
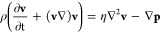
3where vector **ν** and vector **p** (both of which are a function of position and time) are the flow velocity and pressure, respectively; ρ and η are the density and viscosity of the flow, respectively. The left-hand of the Navier–Stokes equation comprises the inertial forces, while the right-hand corresponds to the viscous forces. Here, we introduce an important dimensionless quantity called the Reynolds number (*Re*, expressed in eq 4), which is the ratio of inertial and viscous forces:
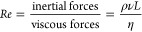
4where *L* is the characteristic length of an object moving in a fluid.

For small-scale devices and organisms (i.e., motile cells, bacteria), *L* is very small (*Re* ≈ 10^–4^), which means that viscous forces rule their motion. A typical analogy of swimming at low *Re* is that a bacteria swimming in water is similar to a person swimming in honey. Considering that inertia forces are negligible in the low *Re* regimes, the Navier–Stokes equation can be simplified as an expression known as the Stokes equation:
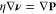
5

**Figure 3:**
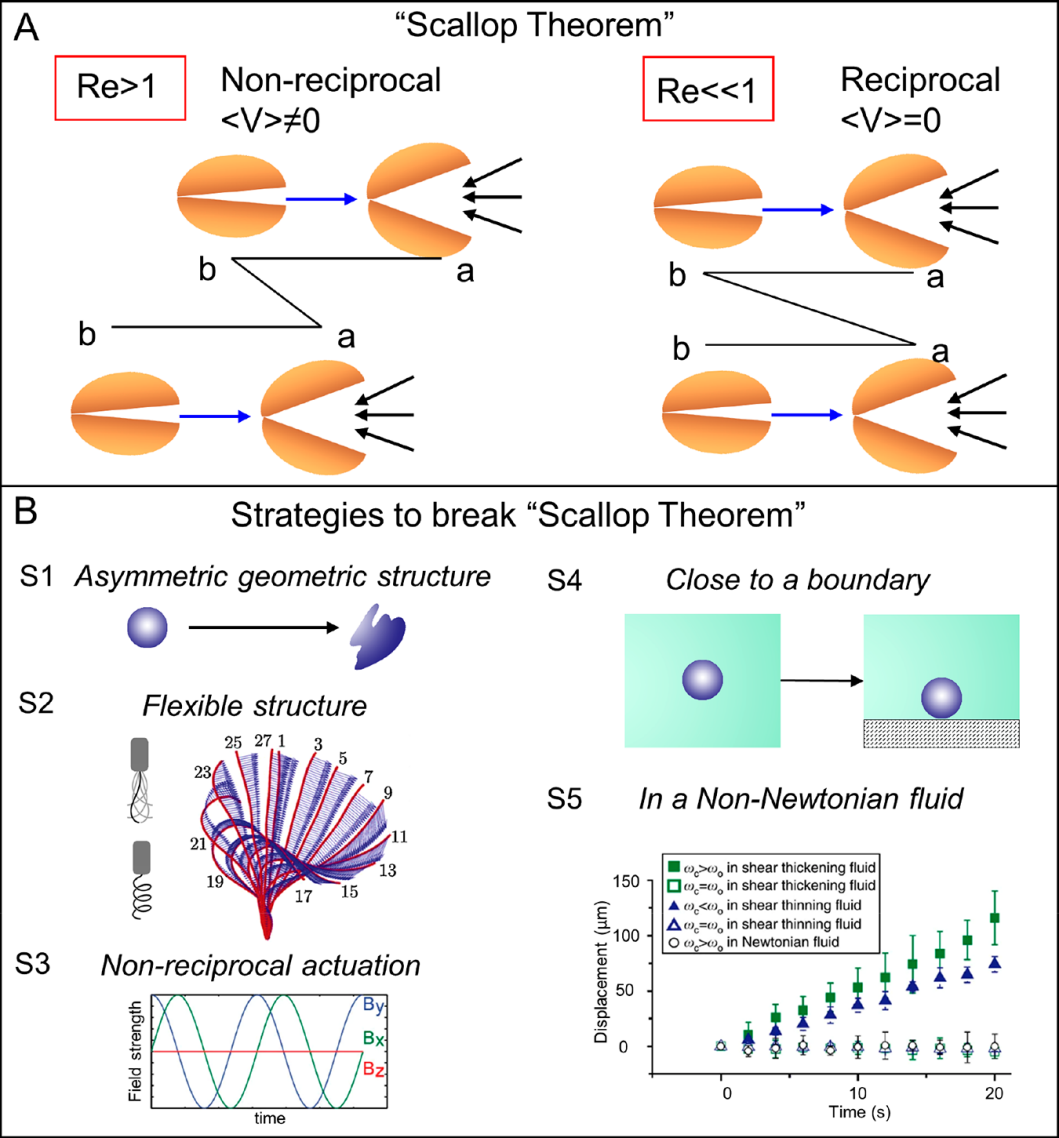
(A) Schematic image of Purcell’s scallop presenting a nonreciprocal motion in a high Reynolds number fluid and reciprocal motion in a low Reynolds number fluid with no net replacement (so-called “Scallop Theorem”^141^). (B) Summary of five strategies (S1–S6) to break the Scallop Theorem to produce an effective movement. S2 is reproduced with permission from refs (137 and 140). Copyright 2014, Brumley et al. This article is distributed under the terms of the Creative Commons Attribution License. S4 is reproduced with permission from ref (142). Copyright 2015 The authors. S5 is reproduced with permission from refs (137 and 140). Copyright 2014 Macmillan Publishers Limited. This is an open access article distributed under the terms of the Creative Commons CC BY license.

Note that this hydrodynamic equation is time-independent, meaning that no net displacement will occur after completing a cyclic process no matter if the speed of the swimmer is fast or slow. In other words, the resultant fluid flow exhibits instantaneous and time-reversible features. This is the so-called “Scallop Theorem,” as introduced by the Nobel laureate Purcell (Figure 3A). At low Reynolds number, a microscopic scallop can only perform back and forward movement (i.e., reciprocal motion). Once the actuation energy (such as a magnetic field) is removed, its motion is immediately halted due to the lack of inertial forces. Importantly, to generate a nonreciprocal translatory movement to execute tasks such as cargo delivery, Figure 3B summarizes some strategies employed to break Purcell’s Scallop Theorem. The first method involves fabricating a small-scale robot with an asymmetric shape such as a tubular,^126^ helical,^60,127,128^ fish-like,^129^ annelid-worm-like,^130^ tadpole-like,^131^ bullet-shaped,^22^ star-shaped,^132^ or even random-shaped^133,134^ structure. In addition, an asymmetric shape (e.g., carpet,^135^ ribbon^56^) can also be formed by self-assembling colloid particles with a symmetric shape based on collective behavior.^90^ A second approach consists of creating a micro- or nanostructure containing a flexible component, for example, a flexible tail, which can mimic the flagellum of a microorganism.^81,136^ Velocity distribution (indexed by frame number of a video sequence) of a single beating flagellum or cilium from a cell or a microorganism during one cycle^137^ indicated the generated traveling-wave motion (see Section 3.2) is nonreciprocal. Incorporating flexible components in between rigid structures to create multilink micro or nanoassemblies is also another possibility, which will be further discussed in Section 4.4. A recent strategy consists of integrating motile flagellated microorganisms and cells with magnetic micro and nanostructures to create biohybrid MagRobots (see Section 4.5). A third approach entails the use of a nonsymmetric actuation magnetic field. For example, a symmetric small structure can exhibit a translational motion by means of a traveling-wave^138^ or a cilia-beating motion mechanism^139^ under a nonsymmetric actuation field. The fourth approach is based on actuating magnetic small-scale devices in the proximity of a boundary (e.g., wall, interface) to break the spatial symmetry. The motion mechanism based on this method is called “surface-assisted propulsion”, which will be discussed in Section 3.3. All these symmetry-breaking strategies evade the constraints of the famous Scallop Theorem.^100^ Note that the Scallop Theorem only applies to Newtonian fluids. Time-reversible reciprocal locomotion can still generate an effective propulsion in non-Newtonian fluids (e.g., blood, saliva, mucus).^140^

## 3 Actuation and Mechanisms of Magnetic Robots

Compared with macroscale motile robots, micro and nanoscale robots experience totally distinctive hydrodynamics. Hence, they exhibit distinctive assorted motion behaviors. A good understanding of various propulsion mechanisms is the basis for the design of propulsion microsystems including the shape and architecture of micro and nanorobots as well as the configuration of the magnetic field. The designed propulsion system must be able to overcome various resistive forces in the micro and nanodomains to realize the motion of small-scale robots effectively. The translational mechanisms of magnetic miniaturized machines could be broadly divided into three types: (a) corkscrew motion, (b) undulatory motion (i.e., traveling-wave motion), and (c) surface-assisted propulsion (i.e., surface walker).

### 3.1 Corkscrew-like Motion

**Figure 4:**
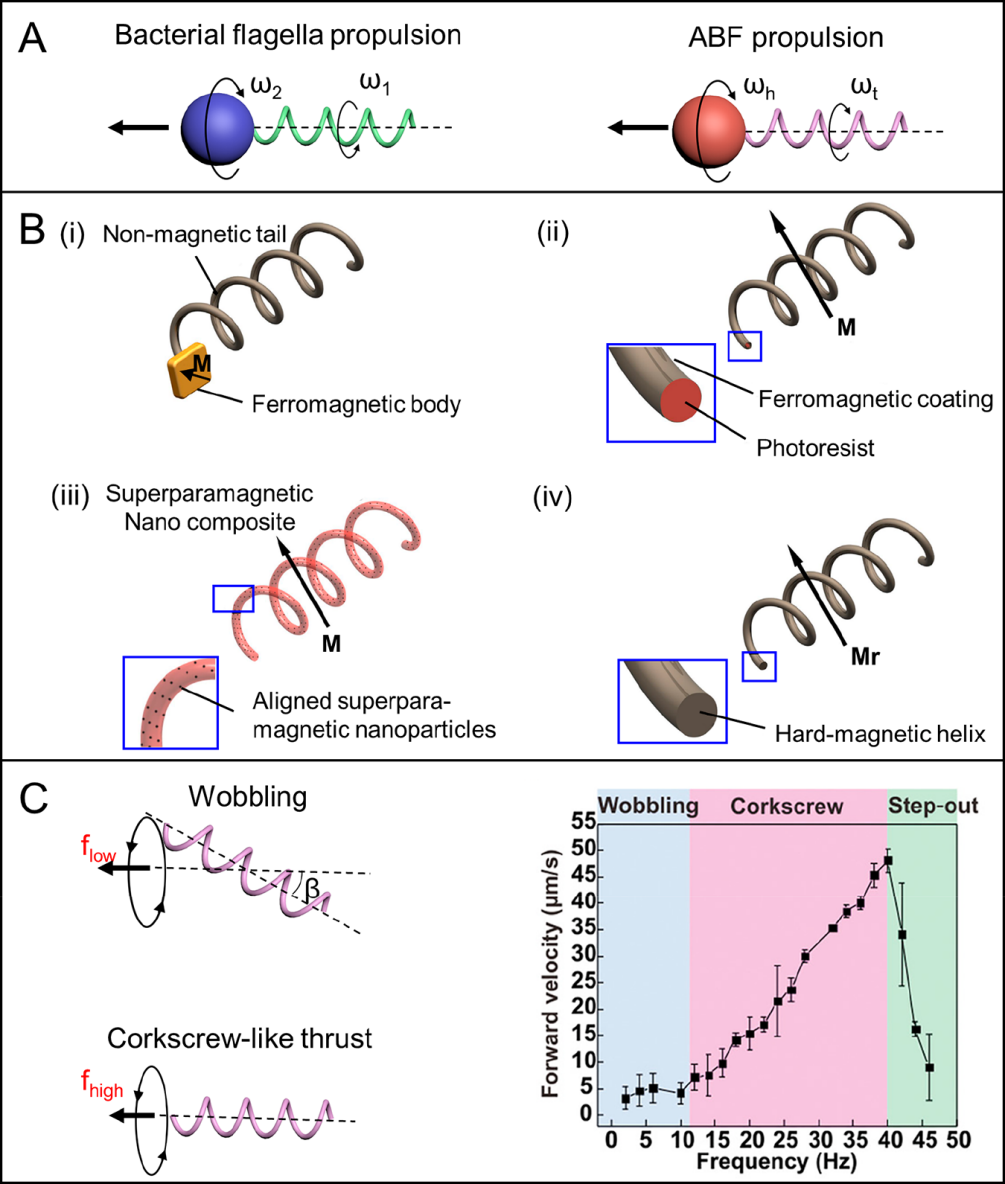
Flagellar-based propulsion mechanisms. (A) Rotation of bacterial flagellum at frequency ω_1_ through rotary motor inside and a counter-rotation of the head at frequency ω_2_, while head and tail of ABF rotate in the same direction. (B) Typical types of magnetic ABFs. Reproduced with permission from ref (149). Copyright 2018 WILEY-VCH Verlag GmbH and Co. KGaA, Weinheim. (C) Field frequency-dependent ABF movement: ABF wobbles with a wobbling angle at low frequency; wobbling movement transforms into corkscrew-like swimming; then the wobbling decreases to zero at high rotational frequencies. Example of frequency-dependent propulsion of MOF-based helical swimmers. Reproduced with permission from ref (155). Copyright 2019 WILEY-VCH Verlag GmbH and Co. KGaA, Weinheim.

In nature, many microorganisms can coordinate their propulsion and orientation behaviors according to external stimuli with a motile appendage called a flagellum. Eukaryotic cells (e.g., spermatozoa) can produce a traveling-wave motion by making use of a flexible beating flagellum. In contrast, prokaryotic cells can perform a corkscrew-type motion by rotating their helical flagella. Bacteria (e.g., *E. coli*), as a representative of prokaryotic organisms, rely on the rotation of flagella for swimming. The flagellum, containing a basal body, a hook, and a filament, is the fundamental organelle for bacterial motion. There is a reversible motor inside the basal body controlling the rotation of the flagellum. The flagellum can not only trigger reorientation of the organism but also make them move forward and back. When the flagellum rotates in one direction with an action frequency ω_1_, the cell body counter-rotates with the reaction frequency ω_2_ (ω_2_ and ω_1_ are not equal) to balance the produced torque (Figure 4A). Inspired by the bacterial flagellum for efficient movement, man-made helical micronanomachines, known as artificial bacterial flagella (ABF),^143−146^ have been developed and investigated. Although there is no motor in the ABF system, external rotating magnetic fields provide a similar function for generating the rotation.

As discussed earlier, a MagRobot will align its easy magnetization axis parallel with the direction of a local homogeneous field upon experiencing a magnetic torque in that magnetic field. A continuously applied torque to a micro/nanoobject under an external rotating field gives rise to the rotational movement of the body. For artificial magnetic micromachines containing chiral helices, a steady rotation around their helical axis can be effectively converted into nonreciprocal translational motion, with the direction parallel with the rotating axis of a two-dimensional planar rotating field. At the same time, the tail and head (sometimes it has no head) of ABF perform the same (clockwise or counterclockwise) orientation. This is distinct from bacteria, whose head and tail rotate in the opposite orientation. If the ABF consists of a single rigid body, then the head and tail will rotate with the same frequency (ω_h_ = ω_t_). Moreover, the progression direction (forward or backward) can be easily inverted by reversing the direction of rotation (i.e., clockwise or counterclockwise) of an applied magnetic field. In the magnetically actuated ABF system, similar to other magnetically controlled systems, magnetic materials are required in order to respond to the external field. Widely used ferromagnetic materials include Ni, Co, and Fe, while the frequently applied superparamagnetic materials include Fe_2_O_3_ and Fe_3_O_4_. Up to now, various types of ABF systems have been investigated.^147,148^ Some typical examples are shown in Figure 4B.^149^

Many factors play a critical role in the movement of magnetic helical microswimmers such as solution properties (e.g., fluid viscosity, ion strength), geometrical parameters (e.g., helix pitch), surface characteristics (e.g., surface wettability,^150,151^ roughness), magnetic field properties (e.g., frequency, intensity, rotating, or oscillating field), magnetization properties of magnetic materials, head/tail shapes, mechanical properties (e.g., rigid or flexible), and boundary condition (e.g., wall). The simulation demonstrates that helical swimmers exhibit the highest propulsion efficiency when the pitch angle is about 45°.^152^ The optimal magnetization direction for helical microrobots is perpendicular to the helical axis in order to maximize the applicable magnetic torque around the axis. The motion mode and velocity of ABF are strongly associated with the applied field frequency. As shown in Figure 4C, at low frequency rotating magnetic fields (typically below several Hertz), a wobbling motion occurs when the axis of the helical MagRobot cannot align with the direction of the local field.^153,154^ As the rotating field frequency is enlarged, the wobbling angle decreases from 90° to zero, where a wobbling angle of zero corresponds to the rotation along the long axis with a direct corkscrew-like thrust. (Ratio of viscous to magnetic torque (i.e., Mason number), helix angle, and helical size can also bring about shrinkage of the wobbling angle of helical MagRobots under temporal–periodic torques.^49^ In the corkscrew-like motion region (also denoted as “synchronous” region), the translational velocity of helical MagRobots increases with the increased applied rotation frequency of an external magnetic field, performing a synchronous and linear relationship. Further increase with respect to a critical field frequency results in a decrease of the swimming velocity, which is attributed to the fact that the magnetic torque is not sufficient to maintain a synchronous relationship between the magnetic moment and the applied rotating magnetic field. The critical frequency is called the “step-out frequency”.^155^

Surface chemistry also influences the motion of helical MagRobots. Recently, it has been reported that magnetically driven helical microswimmers with hydrophobic surfaces possess larger step-out frequencies and higher maximum translatory velocities at low Reynolds numbers in comparison with those with hydrophilic surfaces.^156^ The increase in hydrophobicity of the swimmer surface causes an increase in both the step-out frequency and the maximum forward velocity in a nonlinear mode due to the interfacial slippage. Importantly, the forward velocity of ABF is independent of their surface wettability when MagRobots are manipulated below their critical frequency. A 3D oscillating magnetic field, created by the combination of DC magnetic field B_*xy*_ and oscillating B_*z*_ field, can only cause the reciprocal back-and-forth motion of a helical microswimmer. When symmetry is broken by placing the microswimmer near a surface, the rocking motion results in a net displacement. Moreover, the asymmetric helix (with polystyrene head and helical Co/SiO_2_ tail) exhibits much larger displacement than a nearly symmetric helix without a head under similar experimental conditions.^157^ The viscosity disturbance in different solutions results in the difference of precession angle (i.e., wobbling angle) of helical MagRobots when the applied frequency of the rotating field is smaller than the step-out frequency. Taking advantage of this feature, the detection of instantaneous orientations (i.e., wobbling angle) of MagRobots provides an innovative approach to evaluate the viscosity of the local medium with high spatial and temporal accuracy, which makes ABF a novel prototype for mobile viscometers.^158^

### 3.2 Traveling-Wave Locomotion/Ciliary Stroke Motion

**Figure 5:**
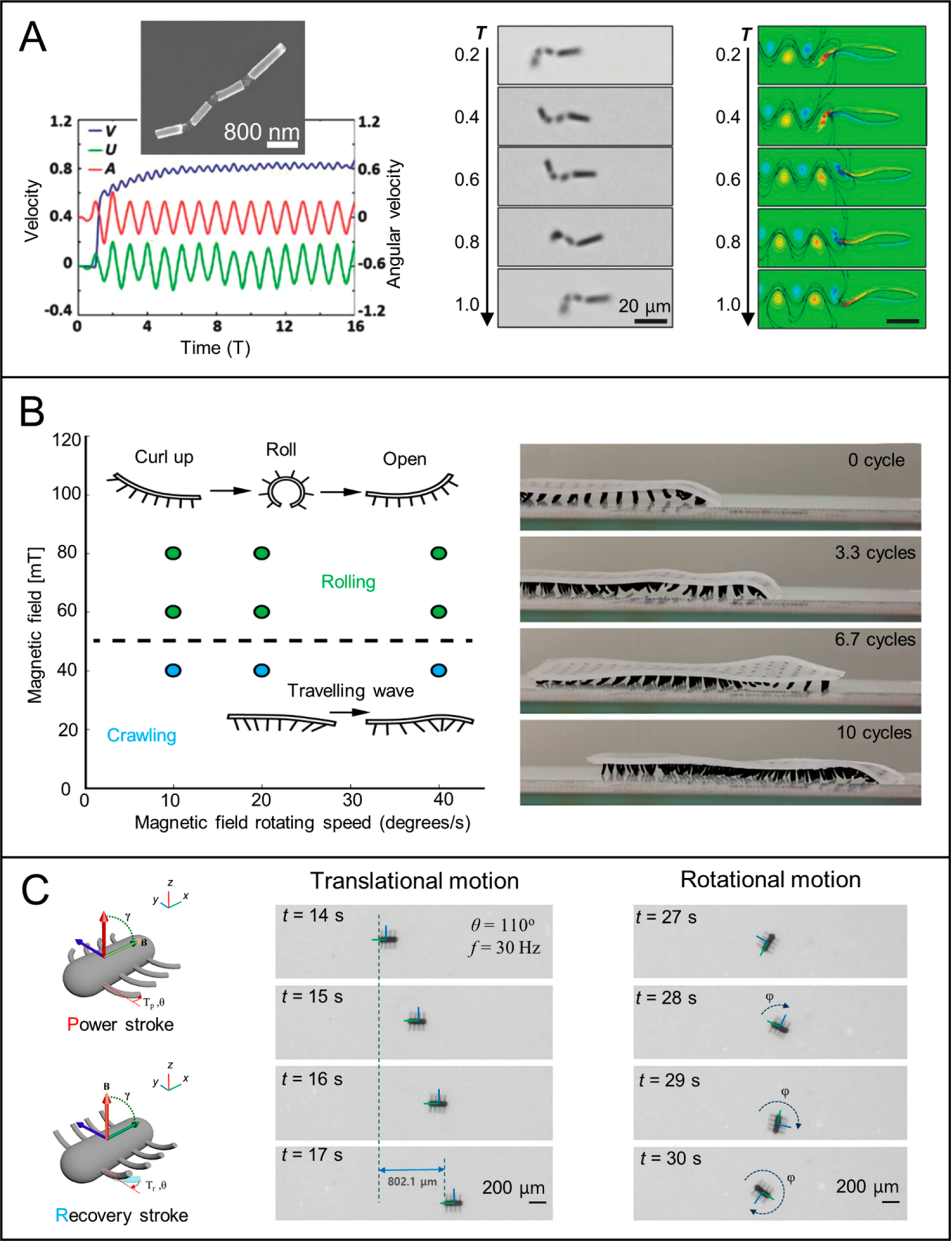
Flagellum-based locomotion of magnetically actuated robots. (A) Motion of Au–Ag–Ni–Ag–Ni–Ag–Au multilink nanowires with flexible silver hinges under a planar oscillating magnetic field. Reproduced with permission from ref (129). Copyright 2016 WILEY-VCH Verlag GmbH and Co. KGaA, Weinheim. (B) Multiple locomotion modes of millipede-like soft robots. Reproduced with permission from ref (160). Copyright 2020 The Authors. (C) Ciliary stroke motion of artificial micromotors. Reproduced with permission from ref (139). Copyright 2016 The Authors.

Both traveling-wave propulsion and metachronal-wave propulsion, inspired by the flagella and cilia of eukaryotic cells, respectively, are capable of breaking temporal symmetry to overcome the Scallop Theorem and generate an effective net displacement. Because short and rigid nano/microrobots can only generate very limited net propulsion due to the reciprocal nature of an oscillating movement, the presence of an elastic component is crucial for achieving traveling-wave propulsion. However, net displacement can also be hampered if the motor is too long and flexible due to the increase of drag force. Hence, the size and elasticity must be taken into consideration in terms of design. Traveling-wave propellers have been created either by incorporating elastic tails (e.g., a chain of paramagnetic beads using DNA as the soft hinge^159^) to a rigid head or by utilizing multilink nanowires connected by flexible segments (e.g., soft silver nanowire,^3^ elastic polymeric nanocylinders composed of multiple bilayers of polyallylamine chloride and polystyrenesulfonate^97^). The thrust from the backward-traveling wave generated by the undulatory motion of multilink artificial microswimmer, consisting of two magnetic nickel segments, two gold segments, and three soft silver, hinges upon the application of an oscillating magnetic field. Periodic mechanical deformation triggered fish-like locomotion at the microscopic level (Figure 5A).^129^ Other traveling-wave motion of wire-like MagRobots driven by an oscillating field can be found in Section 4.4.

Although the metachronal wave, which is produced by the oscillatory locomotion of ciliated protozoa through hydrodynamic interactions, can also drive an effective nonreciprocal movement. Because of the complexity of manufacturing these structures at micro- and nanoscale, only millimeter-scale (not nanoscale or microscale) robot systems that mimic the metachronal-wave movement of cilia have been reported (Figure 5B).^160^ To date, one artificial cilia-like magnetic microarchitecture, as the exclusive example with regard to the simple ciliary stroke motion, has been fabricated by means of a 3D laser lithography method.^139^ The efficient movement of this microrobot in a fluid environment with a low Reynolds number was powered by the net propulsive force from the beating locomotion of cilia and its position and orientation can be precisely controlled by on–off fields with designated angle (Figure 5C).

### 3.3 Surface-Assisted Motion

**Figure 6:**
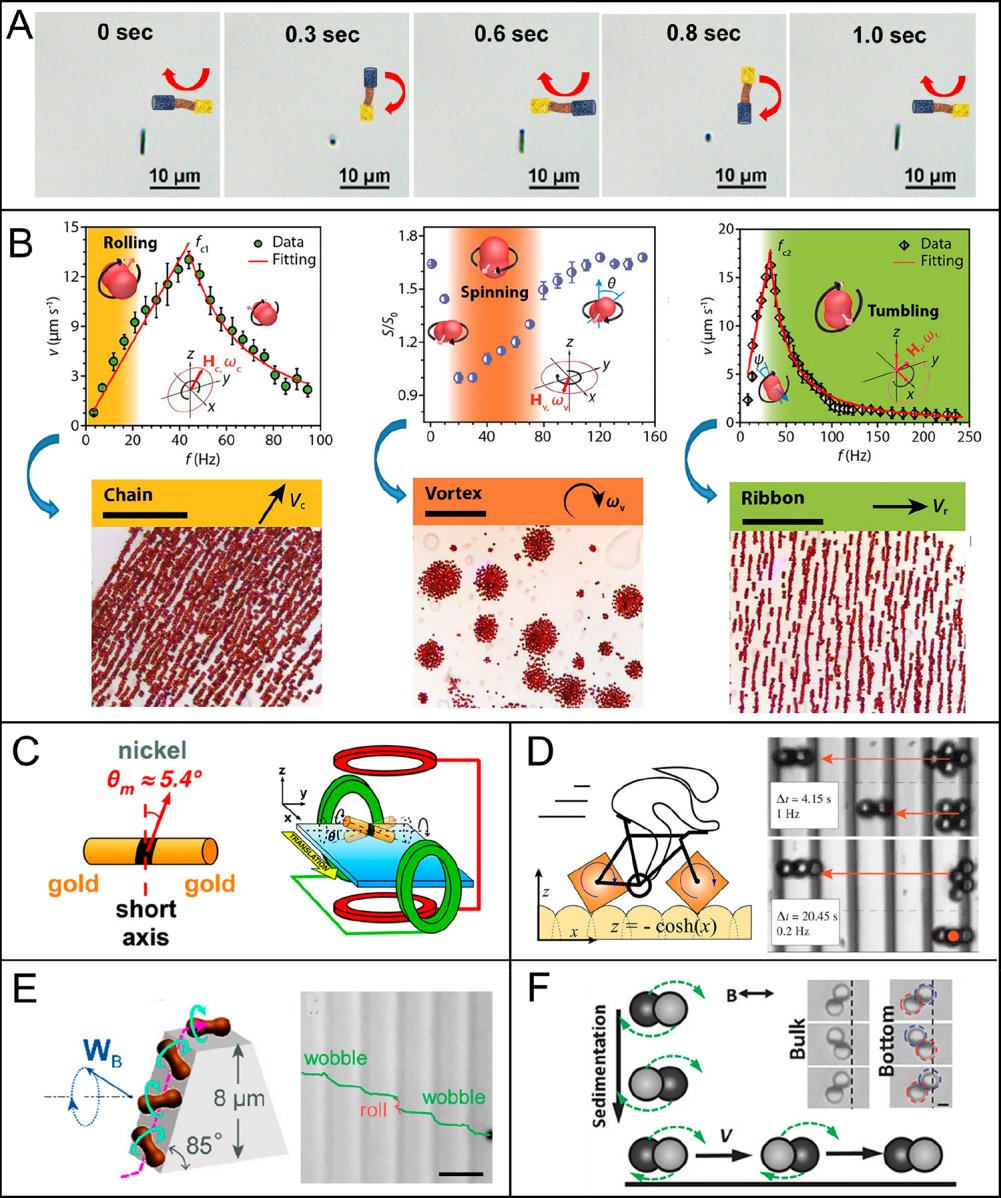
Propulsion mechanisms for surface walkers. (A). Surface-assisted motion of an Au–Ag–Ni nanowire. Reproduced with permission from ref (173). Copyright 2020 American Chemical Society. (B) Motion mode transformation of hematite peanut-shaped microrobots among rolling mode under a *yz*-planar rotating field, spinning mode under an *xy*-planar rotating field, and tumbling mode under a conical rotating field; Swarming patterns of chain, vortex, and ribbon morphologies, respectively. Reproduced with permission from ref (56). Copyright 2019 The Authors, some rights reserved; exclusive licensee American Association for the Advancement of Science. (C) Magnetic coil arrangement and advection of Au/Ni/Au nanowire in kayak motion mode. Reproduced with permission from ref (168). Copyright 2017 The Royal Society of Chemistry. (D) Smooth translation motion of square-wheeled bicycles on bumpy roads and separation of diamond and square μwheels on the textured surface. Reproduced with permission from ref (169). Copyright 2019 The Authors, some rights reserved; exclusive licensee American Association for the Advancement of Science. (E) Schemes of a peanut-shaped motor climbing up a steep slope with the height of 8 μm via a wobbling mode and trajectory of the MagRobot climbing up and down a steep slope. Reproduced with permission from ref (170). Copyright 2018 American Chemical Society. (F) SEM image of a microdimer and its motion in bulk liquid and near a boundary. Reproduced with permission from ref (172). Copyright 2018 WILEY-VCH Verlag GmbH and Co. KGaA, Weinheim.

Apart from breaking the symmetry from the geometrical point of view, another strategy to overcome the Scallop Theorem and induce translational movement is to introduce a physical boundary to break the spatial symmetry. Such locomotion can be achieved by magnetically actuating a magnetic micro- or nanostructure when it lies in the proximity of a surface/interface^161^ or a wall in a liquid at low Reynolds number, or even a dry surface.^162^ The micro and nanorobots based on this “surface-assisted locomotion” mechanism are called “surface walkers” or “surface rollers.” Figure 6A exhibits a typical forward locomotion mode of a surface walker. Many magnetic micro and nanostructures have demonstrated such surface-assisted propulsion including (but not limited to) nanorods, dimers, assembled colloids, microtubes, and Janus particles. Simulations and experiments have confirmed that the dynamics and motion mechanism of surface walkers are governed by the boundary features (slip or nonslip), the degree of confinement (e.g., single or multiple confining boundaries, the distance of a MagRobot from the nearby boundary), fluid properties (e.g., finite inertia^163^), magnetic fields (e.g., configurations, frequency, strength), and others. The presence of a boundary modifies the hydrodynamic stresses on self-propelled nano/microrobots, resulting in a change in their orientation, velocity, trajectory, and even hydrodynamic bound states.^164^ Stronger frictional forces near a nonslip confining boundary (wall or surface) can drive microdevices to move forward, resulting in a larger net displacement compared with those in proximity to a smooth boundary. Hydrodynamic interactions can create stable finite clusters (“critters”) from an unstable front that is generated from the press of fingers.^165^

Motion modes of surface walkers are frequency- and field type-dependent. CoPt semihard magnetic nanowires experience the motion transformation from tumbling to precession and then to almost rolling near a surface boundary by raising the frequency of the applied planar rotating field. In the tumbling region, the *y*-axial translational velocity of nanowires synchronously increases with the field frequency regardless of the applied magnetic moment. In the procession region, the velocity still slowly increases and then decreases after reaching the maximum. The decrease of speed is ascribed to a decline of the precession angle, resulting from the change of motion configuration.^166^ Transformation of the motion mode can also occur in hematite peanut-shaped microrobots by using different magnetic fields, including a 1D oscillating magnetic fields (oscillating mode), *yz*-planar rotating magnetic field (rolling mode), *xy*-planar rotating magnetic field (spinning mode), and conical magnetic field (tumbling mode) corresponding to the collective configuration of liquid, chain, vortex, and ribbon, respectively (Figure 6B). A 2D vortex can be self-assembled by rotating magnetic colloids in a plane parallel to the interface; however, such a vortex cannot produce net displacement. On the contrary, net displacement occurs in rolling mode and tumbling mode once a boundary is present. Taking the chains with rolling mode as an example, net displacement along the *x* axis can be generated when the assembled magnetic chains are subjected to a *yz*-plane rotating field. In other words, the rotational motion of microrobots in a plane perpendicular to a nearby boundary can lead to nonreciprocal propulsion. Similar to the artificial bacterial flagella, the velocity of the individual peanut-shaped microrobots as well as that assembled chains (e.g., trimer and pentamer) linearly increases with applied frequency when the actuation frequency is below the step-out frequency. Above the step-out frequency, the increase of the rotating field’s frequency causes a decrease of the microrobots’ velocity owing to the considerable rise of liquid-induced viscous torque. In addition, the velocity of assembled chains is dependent on the number of microrobots composing the chains. Most importantly, collective formations and locomotion can be manipulated by a magnetic field in a programmable and reconfigurable fashion, providing versatile collective modes to meet multitasking requirements in complicated biological systems.^56,125,167^ Magnetic microkayaks demonstrate processing motion in a double-cone rotating way, similar to the movement of a paddle, when placed in proximity to a solid surface under the rotating fields with kilohertz frequency (Figure 6C).^168^

In comparison with flat surfaces, research of magnetic nano/microrobots on topographic surfaces is more challenging but more intriguing. Inspired by smooth-riding bicycles containing square-shaped wheels, utilization of a microroad with periodic bumps lead to 4-fold intensification in forward velocity of microwheels (μwheels) owing to the nonslip rotation of entire wheels. Because of the velocity difference between diamond μwheels and square μwheels on topographic surfaces, the separation of isomeric μwheels by symmetry can be fulfilled (Figure 6D).^169^ For surface walkers, climbing over a barrier is also possible by taking advantage of surface physics. A peanut-shaped hematite micromotor with its magnetic moment vertical-aligning with the long axis can achieve rolling movement under a rotating magnetic field and wobbling movement under a conical rotating field. The magnetically actuated MagRobot can climb up and down a steep slope with a height of 8 μm through the wobbling motion mode. By combining rolling motion mode and wobbling motion mode, the MagRobots can be utilized to deliver and release cells to an appointed place and form complex cell patterns under the control of a magnetic field in a contactless fashion (Figure 6E).^170^ Except for these artificial barriers, magnetic actuation of MagRobots on the uneven surface of biological tissue (i.e., *ex vivo* swine bladder) was investigated by Zhang’s group.^171^ In addition to a rotating field, an oscillating magnetic field can also be adopted to actuate the translational movement of a surface walker. Under an oscillating field, microdimers consisting of Ni-SiO_2_ magnetic Janus microspheres are able to roll on the solid surface after sedimentation treatment. In contrast, no net displacement can be produced when Janus microspheres are returned to the bulk of the liquid by acoustic levitation (Figure 6F).^172^

### 3.4 Application of Magnetic Fields in Other Propulsion Approaches

**Figure 7:**
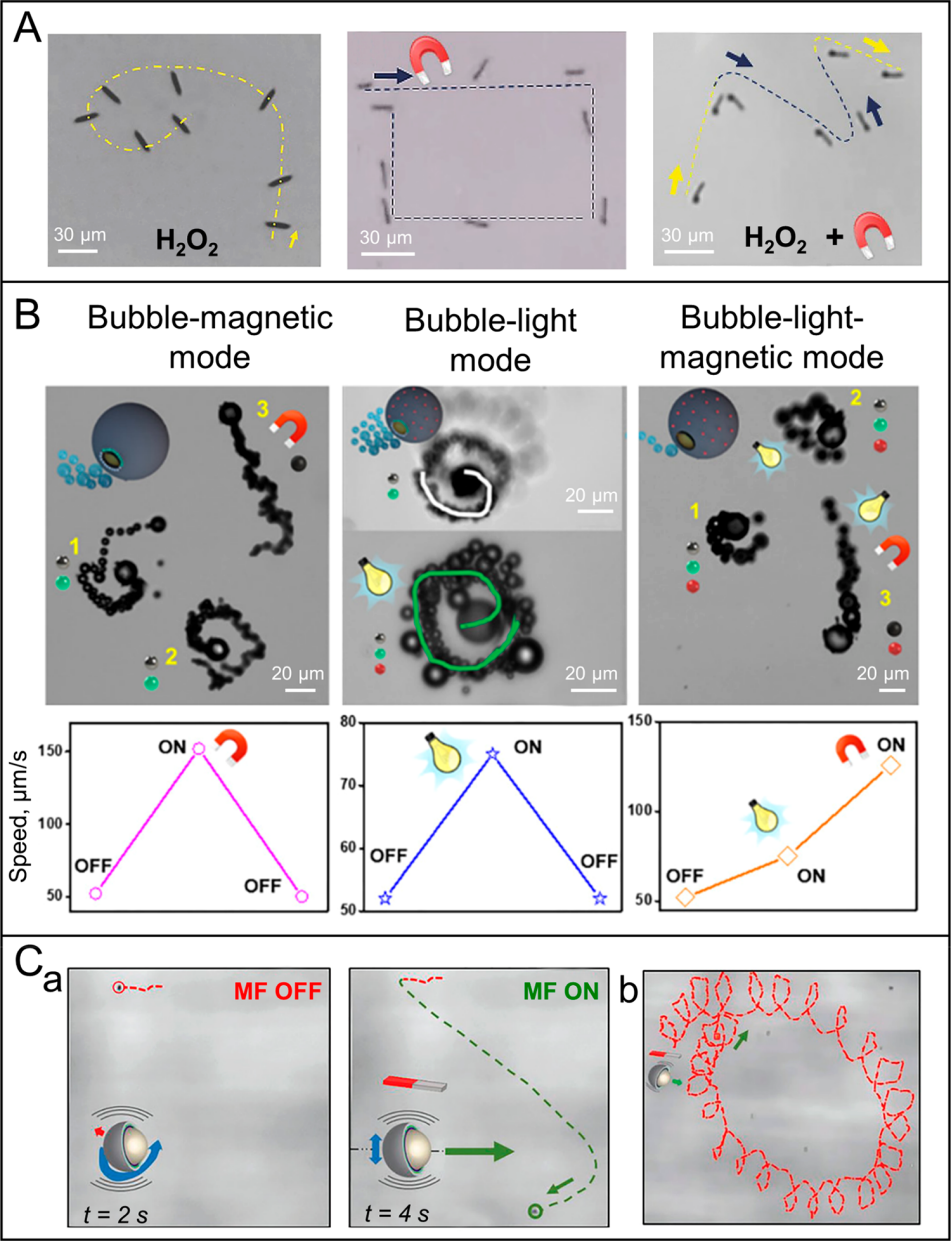
Representative examples of applying magnetic fields to micro/nanorobots actuated by other propulsion sources. (A) Propulsion of a TiO_2_–PtPd–Ni tubular nanomotor by bubbles from the decomposition of chemical fuel, magnetic field, or both. Reproduced with permission from ref (174). Copyright 2016 WILEY-VCH Verlag GmbH and Co. KGaA, Weinheim. (B) Boost of propulsion velocity of a Janus micromotor propelled by dual mode or ternary mode. Reproduced with permission from ref (180). Copyright 2020 American Chemical Society. (C) ON–OFF feature and direction control capacity of the magnetic field for ultrasound-powered Janus micromotors: (a) Propulsion of a single microrobot without and with the application of a static magnetic field; (b) Magnetic navigation of a single acoustic-powered microrobot. Reproduced with permission from ref (181). Copyright 2020 WILEY-VCH GmbH and Co. KGaA, Weinheim.

Approaches such as chemically or photochemically induced propulsion lack the level of control of magnetically driven micro and nanoswimmers, especially in terms of directionality, control over the speed, and ON/OFF motion features. However, chemically and photochemically driven swimmers are very useful for chemistry-on-the-fly applications such as water remediation applications. To provide better controllability on the motion aspects of these chemical and photochemical swimmers, the integration of magnetic components has been widely adopted. For example, a single TiO_2_–PtPd–Ni nanotube^174^ performed autonomous motion through the bubbles generated from the decomposition of hydrogen peroxide (Figure 7A). To control the directionality of bubble-propelled small-scale machines along any predetermined paths, the assistance of other power sources is necessary. After the application of a static magnetic field, the motion direction of those self-propelled nanodevices is controllable. A similar function of orientation control was found in fuel-free light-driven small-size robot systems,^175^ urease-powered nano/micromotors,^176^ cell-powered nanomicromachines,^177^ and acoustically actuated micronanoscale vehicles.^97,178^ Furthermore, the combination strategy can amplify the propulsive thrust by harvesting energies from different sources,^179^ resulting in more efficient task processing capabilities. A Janus microrobot, using three types of nanomaterials as engines, was capable of swimming by bubble propulsion, light-powered propulsion, and magnetic-actuated motion (Figure 7B).^180^ Compared with only bubble-propulsion, the bubble–magnetic dual propulsion mode boosted the velocity of microrobots up to 3 times, while the bubble–light dual mode could increase it up to 1.5 times. Because of the synergetic effect of the three energy sources (i.e., chemical energy, light, and magnetic field), the ternary bubble–light–magnetic mode exhibited a much higher speed than binary bubble-light mode.^180^ By switching on and off a magnetic field, the on-demand control of nanoand microscale robotic systems via braking or accelerating the propulsion process was demonstrated. Obvious growth of velocity was observed in an ultrasound-powered Janus micromotor when a static magnetic field switched from “OFF state” to “ON state” as shown in Figures 7C.^181^ Moreover, the use of external magnetic fields allowed for controlling the directionality to the acoustically driven microrobots.

### 3.5 Magnetic Stimulation of Micro/Nanorobots beyond Motion

**Figure 8:**
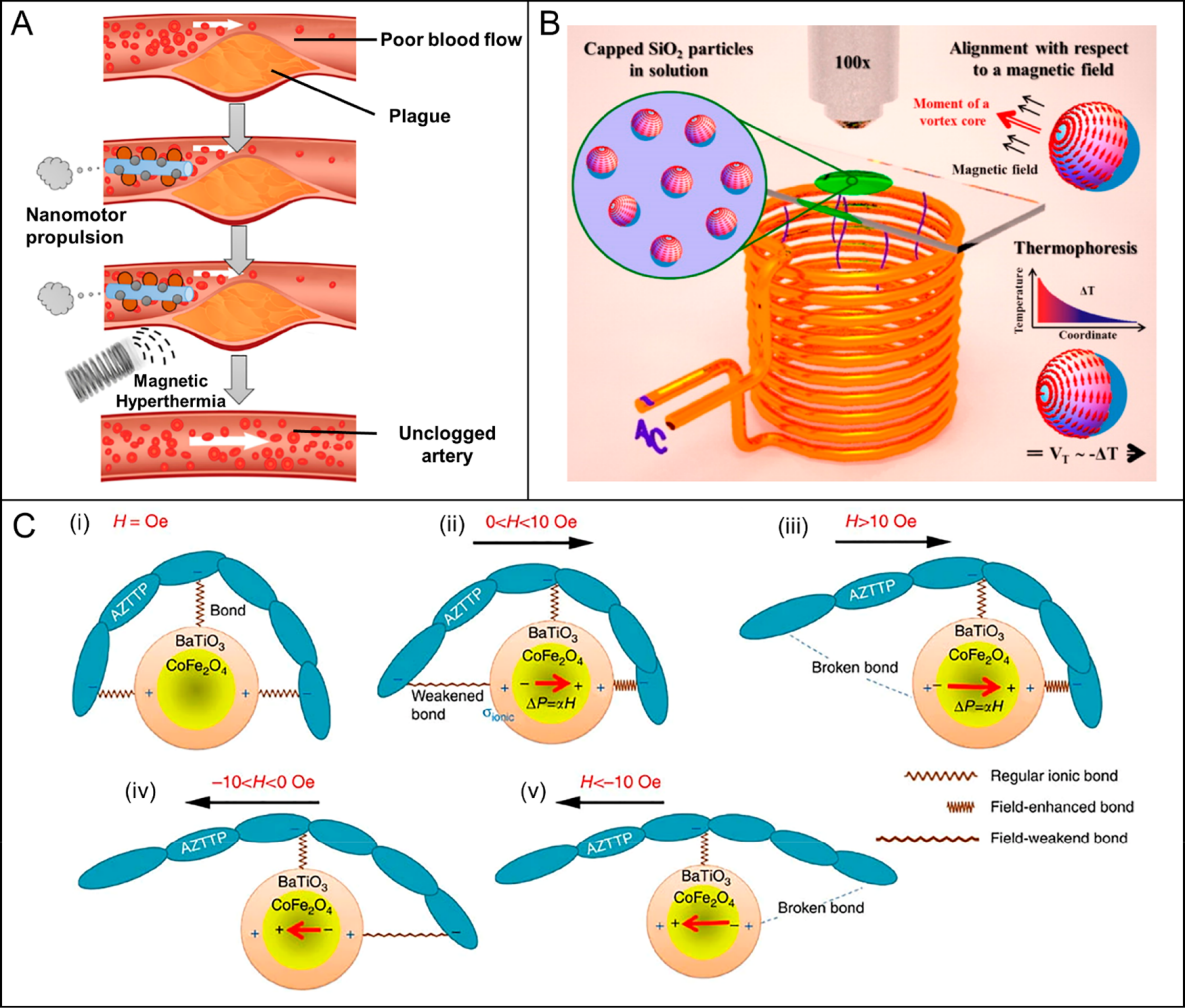
Magnetic stimulation of micro/nanorobots for hyperthermia, thermophoresis, and magnetoelectric applications. (A) Schematic process of removing cholesterol plaque in the blood artery via the magnetic hyperthermia of nanorobots. Reproduced with permission from ref (186). Copyright 2020 Elsevier B.V. (B) Experimental setup of Janus nanorobots for magnetically induced thermophoresis. Thermophoretic force, triggered by the temperature difference, causes the self-propulsion of a Janus particle. Reproduced with permission from ref (124). Copyright 2012 American Chemical Society. (C) Underlying physics of the magnetoelectrically triggered drug (i.e., AZTTP) release process. Reproduced with permission from ref (201). Copyright 2013 Macmillan Publishers Limited.

In addition to direct motion control, magnetic fields can be used as the energy source for triggering hyperthermia,^182^ thermophoresis, and magnetoelectricity. Magnetic hyperthermia refers to the heating of cells, tissues, tumors, or systems to temperatures up to 42 °C by converting magnetic energy into heat radiation.^183,184^ Such function is preferable for treating cancer cells while minimizing damage to surrounding healthy tissues as nanoscale and microscale robots can be externally delivered to the infection site with the assistance of real-time image guidance (e.g., clinical MRI scanner, magnetic particle imaging scanner^185^) and subsequent hyperthermia treatment is localized by only focusing on the tumor tissue. Recently, an approach that combined hyperthermia features with the propulsion force of nanoswimmers has been utilized to clear away plaques in a clogged blood artery. The nanorobots consisted of cellulose nanocrystals, Fe_2_O_3_ NPs, and Pd NPs.^186^ As demonstrated in Figure 8A, the flow of the bloodstream went back to its normal state after the blockage site from animal fat was fully melted and removed. Magnetically induced thermophoresis refers to a self-diffusive motion generated by the local temperature gradient induced by the nano/microrobot itself under an external field. An alternating (AC) magnetic field has been used to heat the spherical Janus robot half-capped with magnetic material (i.e., Fe_19_Ni_81_ alloy), giving rise to self-thermophoretic motion^124^ as shown in Figure 8B. Besides, the high heating power generated by the magnetic field was also reported to trigger a Fischer–Tropsch synthesis.^187^ In this process, the magnetic nanoparticles acted as magnetically induced heterogeneous catalysts.

Magnetic fields can also be used to trigger electric polarization if magnetoelectric materials are incorporated in small-scale motile devices.^188,189^ Magnetoelectric materials are single-phase or composite materials, which become electrically polarized when subjected to an external magnetic field.^188,190^ To operate at room temperature, magnetoelectric materials are usually made by intimately coupling magnetostrictive and piezoelectric components, although certain single compounds, such as bismuth ferrite (BiFeO_3_), exhibit magnetoelectric features at room temperature. When a magnetic field is applied to these materials, the magnetostrictive part changes its dimensions. In turn, the magnetostrictive part stresses the piezoelectric part, which subsequently becomes electrically polarized. Magnetoelectric composites can be processed as bilayered or multilayered composite structures, core–shell architectures, or as particulate matrix composite films.^191^ Because of their ability to generate electric fields in a wireless fashion (i.e., external magnetic fields), magnetoelectric materials integrated into small-scale robots can serve at least two purposes: (a) magnetic navigation due to the responsiveness of the magnetostrictive component to magnetic fields and (b) application of an electric field to the surrounding environment (i.e., electrolytes, cells, tissues) due to the piezoelectric block. Switching between these two capabilities is managed by changing the conditions in which the magnetic fields are applied, for example, by changing the frequency of an oscillating magnetic field or by swapping between gradients (for motion) and oscillating magnetic fields (for triggering the magnetoelectric effect). The delivery of electric fields is interesting for a wealth of applications, especially in the biomedical domain such as cell electrostimulation and differentiation,^192^ electroendocytosis-mediated drug delivery,^193^ irreversible electroporation for cancer treatment,^194^ cell fusion,^195^ or even cell destruction.^196,197^ Magnetoelectric nanorobots or microrobots, despite being less investigated, have been utilized for targeted cell manipulation,^198^ neuronal-like cell differentiation,^13^ and targeted drug delivery.^199^ For instance, a helical microswimmer, incorporating core–shell magnetoelectric nanoparticles (i.e., CoFe_2_O_4_ as the core and BiFeO_3_ as the shell) into a hydrogel matrix was able to induce the differentiation of neuronal cells due to the generation of charges upon magnetic stimulation.^13^ On-demand drug release for killing cancer cells was demonstrated by FeGa@P(VDF-TrFE) core–shell nanowires upon the application of an AC magnetic field because of the magnetoelectric coupling effect.^199^ It is believed that magnetoelectrically induced drug release is caused by the rupture of drug–carrier bonds when the dipole moment triggered by a magnetic field goes beyond the threshold value (i.e., drug–carrier bond strength) and breaks the intrinsic charge distribution on atoms^200,201^ as suggested by Khizroev’s group (Figure 8C).

## 4 Magnetic Robots in the Making: Fabrication Approaches

### 4.1 (Quasi-)Spherical MagRobots

**Figure 9:**
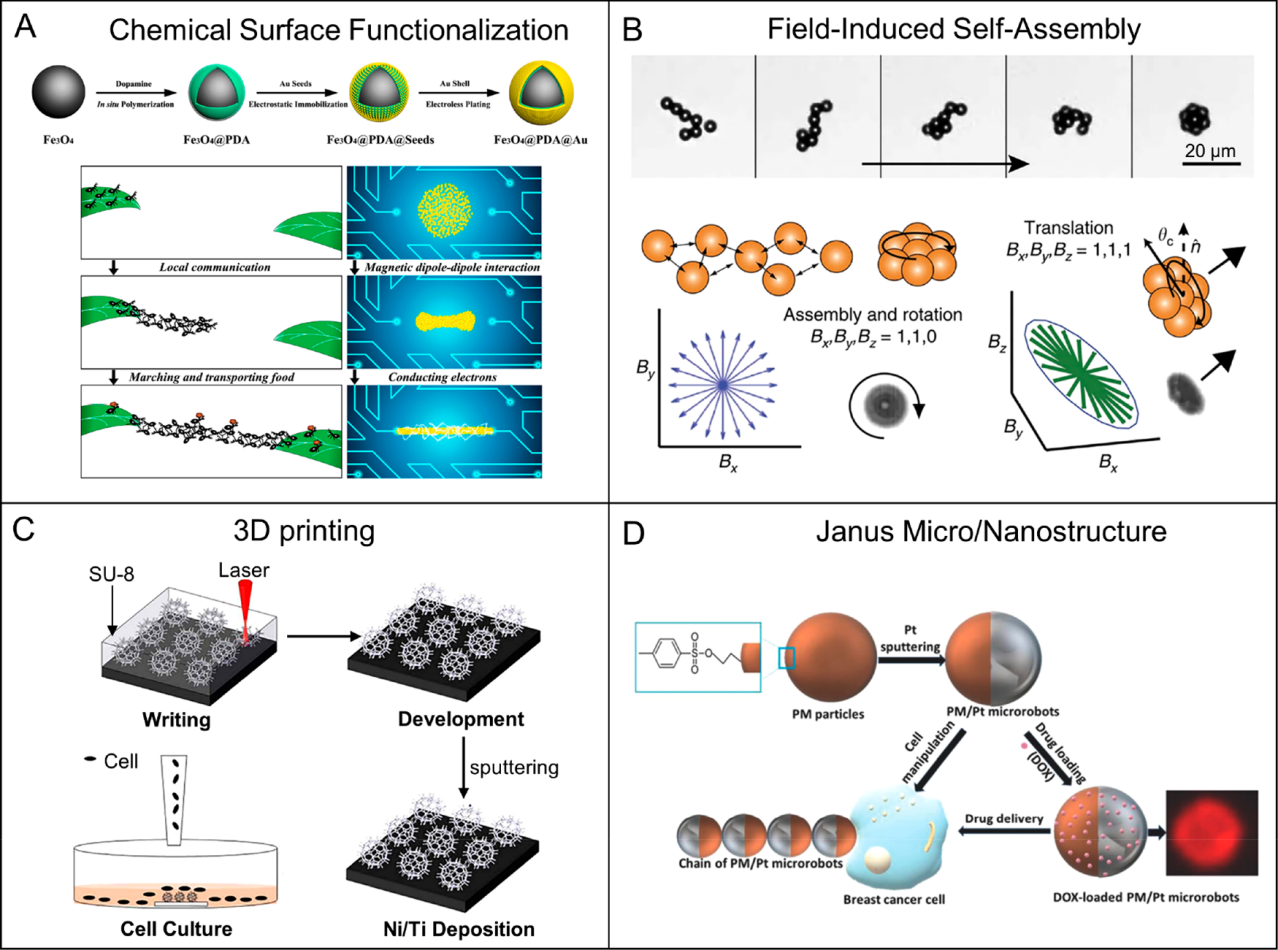
Schematic illustrations of the representative fabrication processes of (quasi-)spherical MagRobots. (A) Fabrication steps of Fe_3_O_4_@PDA@Au MagRobots and formation process of an ant bridge, corresponding to conceptual steps for a reconfigurable microswarm to repair an electrical circuit. Reproduced with permission from ref (212). Copyright 2019 American Chemical Society. (B) Microwheel prepared from the self-assembly of superparamagnetic Dynabeads M-450 Epoxy by rotating field and its field-dependent motion modes: planar rotating magnetic field makes colloids assemble and microwheels spin, whereas 3D oscillating magnetic field makes microwheels roll along the surface. Reproduced with permission from ref (214). Copyright 2019 The Authors. (C) Fabrication steps of the burr-like microrobots. Reproduced with permission from ref (215). Copyright 2018 The Authors, some rights reserved; exclusive licensee American Association for the Advancement of Science. (E) Fabricating process of PM/Pt Janus microrobots for cell manipulation, DOX drug loading, and delivery. Reproduced with permission from ref (210). Copyright 2018 WILEY-VCH Verlag GmbH and Co. KGaA, Weinheim.

Colloidal magnetic particles have attracted scientists’ attention not only because of their individual properties but also due to an emergently investigated phenomenon called “swarm” or “collective behavior”,^57,202−209^ which is a term inspired by many phenomena in nature such as flocking of birds or team-work behaviors of insects. How to manipulate and actuate a large number of tiny robots with collective behaviors for potential *in vivo* applications, particularly in complex biological media and in a precisely controllable and programmable fashion, is the ultimate objective of scientists. The self-assembled MagRobots not only are capable of loading or unloading defined cargos on command but also transport them to a defined site (e.g., microfluidic system or biological environment), providing great potential for localized therapy and targeted drug delivery^210^ owing to their easy synthesis and versatile multifunctionalities by material design, structure optimization, and surface modification. The collective behavior via colloidal self-assembly presents a rapid, reversible, and programmable bottom-up approach to fabricate MagRobots by employing simple colloidal particles as building blocks. In the presence of a magnetic field, both commercially purchased paramagnetic materials (e.g., μm-sized Dynabeads^135,211^) and experimentally synthesized magnetic colloidal particles can be self-assembled into desired sizes and shapes (such as carpet,^135^ wire,^212^ lasso^211^). Yang et al.^211^ recently reported on superparamagnetic PVA-linked colloidal chains by applying a one-dimensional DC magnetic field with a strength of around 20 mT in the vertical direction to a diluted epoxy-functionalized Dynabeads solution. After the formation of linear chains, a circularly planar rotating magnetic field was operated to transform the chains into a lasso shape. By steering the magnetic field strength and phase lag, lassos can capture cargo through curling behavior and precisely transport it on the ground of a wheel-type mechanism at high velocities. Inspired by ants’ cooperative behavior to create a bridge with their bodies when encountering a vanished or nonexistent road (Figure 9A), Zhang’s group used a self-organized magnetic swarm robotic system as building blocks to form a microswitch to repair broken microcircuits. Each component of the system was made of a conductive gold-coated superparamagnetic Fe_3_O_4_ nanoparticle. Under a programmed oscillating field, these magnetic nanoparticles can self-reconfigure into a ribbon-like microswarm to act as a conductive bridge between two disconnected electrodes. The patterns and behaviors of the swarming MagRobots depend on the amplitude ratio and input oscillating frequency. Moreover, the elongation of the microswarm is reversible by altering the amplitude ratio.^212,213^ By applying an *xy*-plane rotating magnetic field with a few milli-Tesla (mT), microwheels of superparamagnetic beads can be self-assembled (Figure 9B).^214^ For microwheels lying on a surface, magnetic torque generated by a 2D rotating field can only induce a spinning movement of the micromachines without net displacement. After inputting a 3D oscillating field by adding a varied component vertical to the plane of the rotating field, that is, the microwheels were reoriented until they tilted to a surface, they began to translate with a velocity of around 100 μm s^–1^.^214^ Inspired by the rolling motion of neutrophiles on the vasculature walls, superparamagnetic beads can accumulate and roll on the surface of confined boundaries using a combination of magnetic and acoustic fields.^66^

3D laser lithography is among the most popular techniques used to fabricate small-scale robots with desired architecture. Burr-like spherical porous MagRobots were prepared by using a direct laser writing system followed by depositing Ni thin films for magnetic actuation and Ti thin films for biocompatibility via a sputtering system (Figure 9C).^215^ The fabricated microrobots can carry and deliver targeted cells to a predetermined location *in vitro* and *in vivo* under the control of a field gradient. *In vitro* experiments conducted in a microfluidic chip showed that cell-loaded microbots could be transferred along the blood vessel-like microchannel to a predefined area to release cells (i.e., MC3T3-E1 preosteoblasts). These free cells moved toward the tissue chamber through migration channels. *In vivo* experiments conducted on nude mice also confirmed that burr-like magnetic microrobots exhibited excellent cell loading, carrying, and release capabilities. In a similar fashion, Jeon et al. used 3D laser lithography and sputtering to fabricate cylindrical, hexahedral, helical, and spherical MagRobots.^122^ The use of a magnetic field gradient induced the pulling motion of cylindrical and hexahedral MagRobots, while the rotating field caused corkscrew motion for helical MagRobots and rolling motion for spherical microrobots.^122^

Spherical microrobots with Janus structure were fabricated by Martin Pumera’s group (Figure 9D).^210^ The Janus structure, formed by half-covering superparamagnetic polymer particles with catalytic Pt layer, can self-propel due to the catalytic decomposition of hydrogen peroxide and can be steered by an external magnetic field. Polymer particles with a tosyl group-rich surface provided the chance to bind anticancer drugs. In addition to drug loading and delivery, the microrobots could also manipulate cells when they assembled into a chain under magnetic guidance.

### 4.2 Helical MagRobots

Helical architectures, inspired by the flagella of bacteria, enable micronanomachines to convert rotational motion to a translational corkscrew motion by using a low-strength magnetic field in low Reynolds number liquids. Various micro- and nanofabrication techniques have been used to prepare helical micro/nanostructures, including template-assisted electrochemical deposition (TAED),^216^ laser ablation,^217^ direct laser writing and 3D printing,^128,156,218−221^ glancing angle deposition,^127,222^ coiled flow template,^223,224^ biotemplate,^225,226^ and origami-based self-scrolling technique.^60,227^

**Figure 10:**
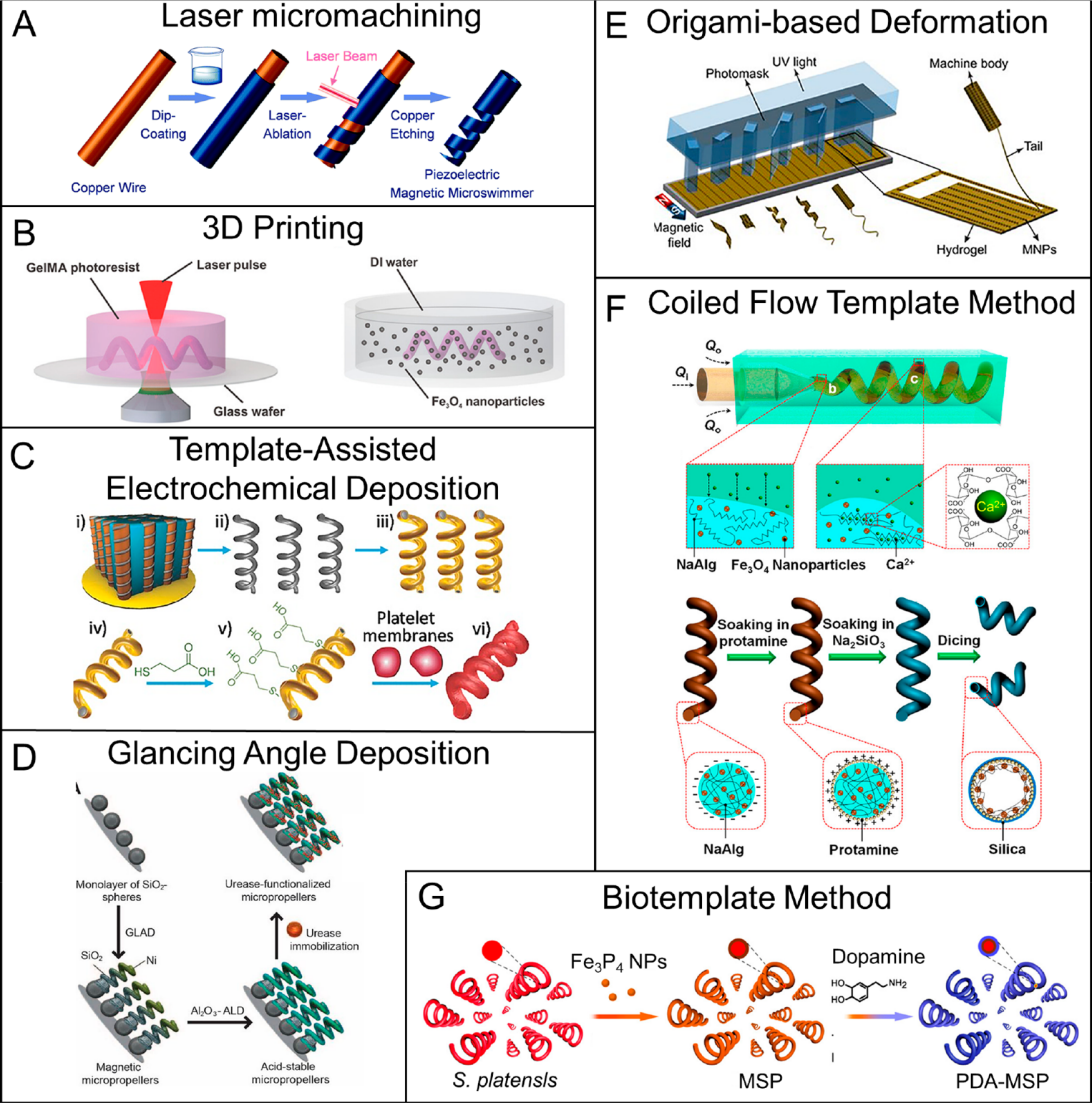
Schematic illustrations of representative synthetic methods for helical MagRobots. (A) Fabrication process of piezoelectric magnetic microswimmers by laser ablation. Reproduced with permission from ref (217). Copyright 2019 The Royal Society of Chemistry. (B) Fabrication of biodegradable helical MagRobots using two-photon polymerization. Reproduced with permission from ref (238). Copyright 2018 WILEY-VCH Verlag GmbH and Co. KGaA, Weinheim. (C) Preparation process of platelet-membrane-cloaked MagRobots by TAED method including (i) Pd/Cu coelectrodeposition, (ii) etching of Cu and collection of helical structures, (iii) deposition of Ni and Au layers, (iv) collection of helical nanostructures, (v) surface modification, and (vi) fusion of platelet-membrane-derived vesicles to the modified surface. Reproduced with permission from ref (216). Copyright 2017 WILEY-VCH Verlag GmbH and Co. KGaA, Weinheim. (D) Preparation steps of acid-stable enzyme-functionalized MagRobots by GLAD. Reproduced with permission from ref (222). Copyright 2015 The Authors, some rights reserved; exclusive licensee American Association for the Advancement of Science. (E) Origami-inspired approach to prepare microswimmers by one-step photolithography. Reproduced with permission from ref (245). Copyright 2019 The Authors, some rights reserved; exclusive licensee American Association for the Advancement of Science. (F) Fabrication of helical microrobots with hollow structures with the assistance of coiled flow template. Reproduced with permission from ref (223). Copyright 2018 American Chemical Society. (G) Fabrication process of biohybrid microswimmers based on *Spirulina platensis*. Reproduced with permission from ref (226). Copyright 2020 American Chemical Society.

Laser micromachining allows the creation of arbitrary 3D structures. Piezoelectric soft MagRobots, which can deliver PC12 cells by employing a rotating magnetic field to induce neuronal differentiation under the stimulus of acoustic waves, were fabricated by Salvador Pané’s group.^217^ Helical MagRobots consisting of piezoelectric polymer matrix and CoFe_2_O_4_ magnetic component were formed by laser ablation of composite film coated on the surface of copper wire by dip-coating method, followed by etching copper wire with acidic ferric nitrate solution (Figure 10A). Steering of helical parameters such as pitch, pitch angle, and the ratio can be achieved by altering the laser spot size, laser motion speed, and rotating speed of copper wire. The helix microstructure can move in a corkscrew manner along its long axis by a rotating field.

3D/4D printing provides a feasible approach to fabricate soft micro/nanorobots with predesigned shapes.^228−236^ Recent reviews give a summary of functional soft robots created by 3D printing^45^ and 4D printing^237^ technique. 3D-printed enzymatically biodegradable soft helical microswimmers have been designed by Pané and co-workers.^238^ Two-photon polymerization (a type of 3D printing technique) was adopted to print photo-cross-linkable gelatin methacryloyl (GelMA) helical microswimmer. To decorate GelMA architecture with Fe_3_O_4_ nanoparticles for magnetic actuation, GelMA microstructures were immersed in a water suspension of PVP-coated Fe_3_O_4_ nanoparticles (Figure 10B). Another work about hydrogel-based biodegradable helical microswimmers with length of 20 μm and diameter of 6 μm was reported by Metin Sitti’s group.^128^ 3D printing of double-helical architecture was realized by two-photon polymerization technique from a precursor mixture of GelMA, photoinitiator, and biofunctionalized superparamagnetic Fe_3_O_4_ nanoparticles. Such double-helical architecture allows these micromachines to host high therapeutic cargo loading and swimming abilities under a rotating magnetic field.

Although template-assisted electrochemical deposition (TAED) has been widely used to fabricate tubular micromotors, this method can also be employed to generate helical architectures.^239−241^ A representative example was demonstrated by fabricating platelet–membrane-cloaked magnetic helical nanomotors in Joseph Wang’s group.^216^ Pd helical microstructures with a length of 3–5 μm were synthesized by coelectrodepositing a Pd/Cu bilayer on an electrochemical platform using a polycarbonate template and followed by selectively etching the Cu with nitric acid. Afterward, Ni/Au thin films were deposited on the surface of the helical nanostructure via the electron beam evaporation method. To make the gold surface negatively charged, surface modification of the magnetic helical microstructures was carried out by overnight incubation of the microrobots with 3-mercaptopropionic acid. Then, platelet-membrane-derived vesicles were adsorbed, bound, and fused onto the negatively charged gold surface by ultrasonic mixing (Figure 10C).

Helical MagRobots can also be produced by glancing angle deposition (GLAD).^242−244^ In this approach, a seed layer, normally created by spreading a monolayer of silica beads on the substrate, is required to function as the nucleation site. Prior to deposition, the seed layer is fixed at a glancing angle with respect to the input vapor flux of a specific material. During the deposition process, a helical silica structure grows starting from an individual seed particle by continuously rotating the substrate. The pitch and chirality of asymmetric helical structures are changeable by adjusting the speed and direction of rotation. Finally, a layer of magnetic material is deposited in the resulting silica helical tail. While this method can batch-produce uniform helical nanostructures, this process is still limited in terms of material selection and shape. To make the magnetic section (i.e., Ni) of helical microstructure stable in acidic solution, helices were covered with an 8 nm Al_2_O_3_ thin film by atomic layer deposition. The stabilized helical micropropellers can be further functionalized with urease (Figure 10D).^222^

Inspired by origami designs, Huang et al.^245^ exploited thermoresponsive gel composites reinforced with magnetic nanoparticles to fabricate microswimmers with various 3D architectures by using a one-step photolithography technique and capitalizing on the self-folding of the hydrogel upon hydration (Figure 10E). During the gel polymerization process, a static uniform field was used to align the encapsulated magnetic nanoparticles. The folding axis direction of the MagRobots was consistent with the alignment direction of the magnetic particles as the swelling was constrained along the reinforcement direction. The produced microswimmers could change their shapes to adapt to local environmental variations in mechanical constraints and osmotic pressure.^245^

Hollow helical microstructures can be obtained by first synthesizing magnetic helical microfibers composed of calcium alginate hydrogel and Fe_3_O_4_ nanoparticles from coiled flow templates in glass-capillary microfluidic devices, followed by biosilicification and dicing process (Figure 10F). The produced microswimmer containing inflexible alginate/protamine/silica shell exhibited good mechanical performance for cargo transport.^223^ Utilization of bevel-tip capillary and syringe pump, heterogeneous core–shell hydrogel microsprings with calcium alginate hydrogel as shell components and functional materials (e.g., magnetic particles, agarose, cell-suspended collagen) as core components were produced.^246^

Because nature provides us with plenty of helical micro- and nanoarchitectures, preliminary attempts to extract the helical xylem vasculature of plants^225^ and Spirulina cyanobacterial green–blue microalgae^247−249^ as templates to fabricate biohybrid helical micro- and nanomachines open a new insight into strategic designs. The advantage of biohybrid small-scale robots is in the biocompatibility and biodegradability characteristics of the biotemplates. Cell-based helical microswimmers can be acquired from multicellular Spirulina via a single cost-effective dip-coating process in superparamagnetic Fe_3_O_4_ solution.^249^ Because of the intrinsic properties of microalgae, the prepared microswimmers allowed for *in vivo* fluorescence imaging without additional fluorescent markers. Moreover, large swarms of microswimmers can be accomplished inside the rat stomach by an external rotating magnetic field with the assistance of imaging.^249^ Model small molecules, as well as biomacromolecules, can be loaded into Spirulina cells by controlling their dehydration and rehydration.^247^ The micromachine loaded with molecular cargo can be magnetically driven in an intestinal tract phantom, thus providing the possibility of targeted molecular delivery for gastrointestinal diseases. By modifying their surface with polydopamine via dopamine self-polymerization (Figure 10G), Spirulina-based magnetic helical microswimmers exhibit an enhanced photoacoustic signal and photothermal effect.^226^ In addition to the above-mentioned helical MagRobots, many other helical architectures have been created.^145,243,250−256^

### 4.3 Flexible MagRobots

Flexible or soft small-sized robots refer to a nanoscale and microscale robotic system completely or partially comprising soft components or architectures that function as carriers, templates, hinges, joints, actuators, sensors, or reservoirs.^257−262^ The utilization of flexible microorganisms to create MagRobots will be discussed in Section 4.5. The advantages of flexible MagRobots are reflected in the following aspects: First, as described in Section 2.4, the integration of a soft segment as a hinge^262^ (see Section 4.4) or as a tail (see Section 3.2), into nano/microrobots can break spatial and temporal symmetries and generate a forward thrust. Second, flexible MagRobots are capable of transforming their configurations/architectures to execute special tasks under the magnetic actuation, such as grasp and release (similar to the function of a hand) of a small-scale object.^263,264^ Third, flexible and soft small-scale robots are more desirable for biomedical applications as these devices are more adaptive in complex biological scenarios, especially in confined, hard-to-reach tissues and vessels of the body when compared with swimmers made from rigid and hard parts.

**Figure 11:**
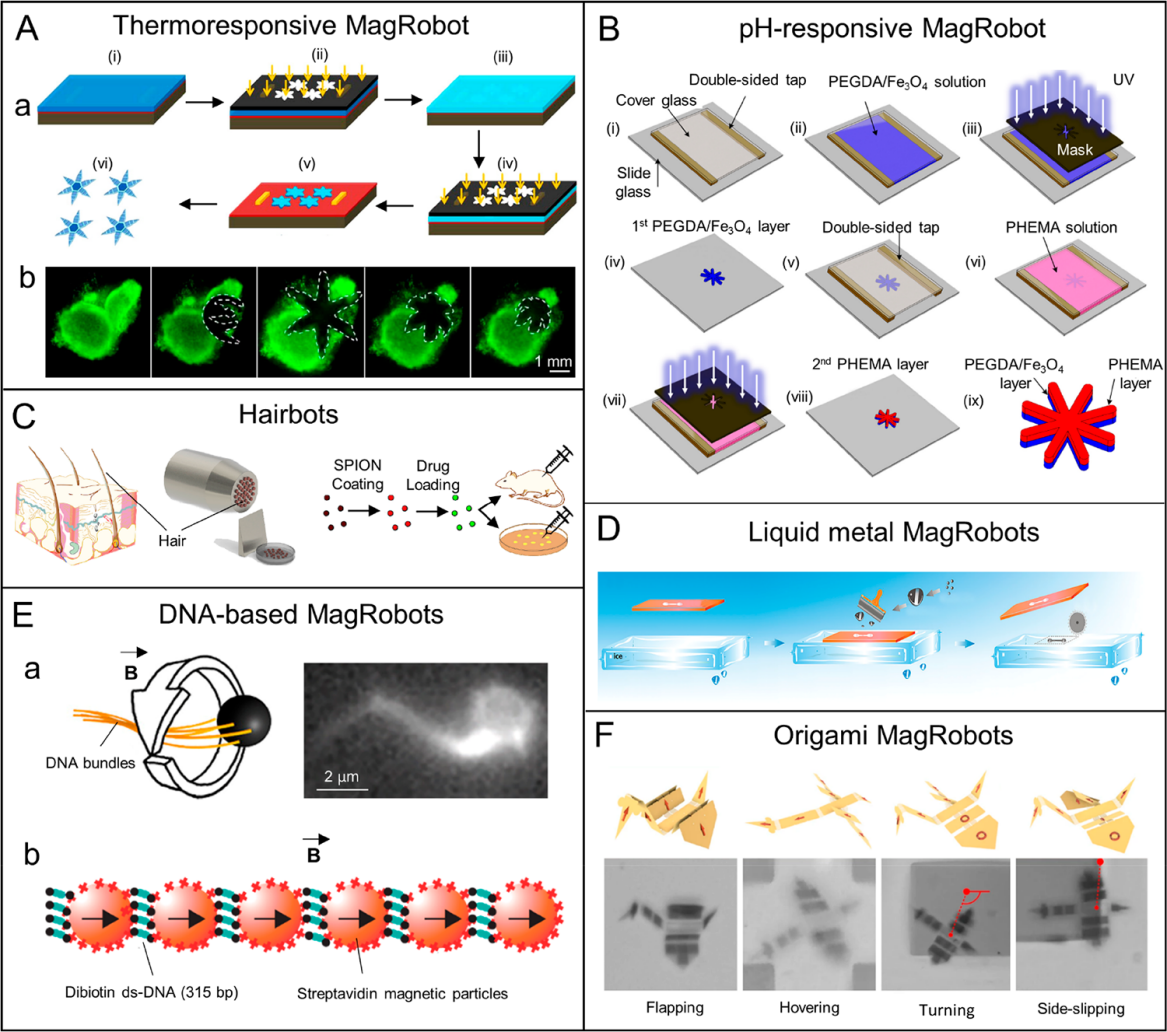
Schematic illustrations of the representative fabrication processes of flexible MagRobots. (A) (a) Fabrication process of temperature-sensitive microgripper including (i) depositing metal alignment markers and spin-coating sacrificial layer and PPF/DEF solution, (ii) cross-linking PPF segments by UV light through a mask, (iii) coating pNIPAM-AAc layer on top of the wafer, (iv) photopatterning the pNIPAM-AAc layer by UV light through a mask, (v) removing uncross-linked chemicals, and (vi) releasing microgrippers from the wafer by dissolving the underlying sacrificial layer in water; (b) Cell capture and excision due to the reversible folding/unfolding behavior of microgrippers in response to temperature. Reproduced with permission from ref (39). Copyright 2015 American Chemical Society. (B) Fabrication procedure of pH-sensitive soft MagRobot. Reproduced with permission from ref (266). Copyright 2016 IOP Publishing Ltd. (C) Formation of hairbots by sectioning a bundle of hair by ultramicrotome and then loading hairbots with magnetic particles and drugs. Reproduced with permission from ref (268). Copyright 2019 Elsevier Ltd. (D) Preparation of liquid metal MagRobots. Reproduced with permission from ref (269). Copyright 2019 WILEY-VCH Verlag GmbH and Co. KGaA, Weinheim. (E) DNA-based flexible MagRobots: (a) Preparation of a hybrid MagRobot with flexible DNA flagella via DNA self-assembly method. Reproduced with permission from ref (270). Copyright 2016 American Chemical Society. (b) Fabrication of a flexible magnetic filament by binding magnetic particles with double-stranded DNA via the specific biotin–streptavidin interaction under a magnetic field. Reproduced with permission from ref (159). Copyright 2005 Nature Publishing Group. (F) Origami-like MagRobots with various shape-morphing modes, mimicking the flapping, hovering, turning, and side-slipping of birds. Reproduced with permission from ref (64). Copyright 2019, The Authors, under exclusive license to Springer Nature Limited.

Soft robots can be constructed with stimuli-responsive polymer materials that enable shape transformations and the realization of other tasks depending on environmental changes (i.e., pH,^265,266^ temperature). For example, PPF/pNIPAM-AAc magnetic microgrippers with pNIPAM-AAc serving as a thermoresponsive swelling hydrogel segment, polypropylene fumarate (PPF) as a nonswellable stiff segment, and Fe_3_O_4_ nanoparticles for the magnetic actuation were prepared by serial photolithographic method (Figure 11A). The thermoresponsive soft self-folding microgrippers could be directed or retrieved to the desired location under the magnetic field to execute their tasks (e.g., to load or release therapeutics) in response to temperature stimulus at around physiological temperature without the need of wires, batteries, or other sources.^39^ Similarly, another thermoresponsive soft microrobot was manufactured and employed for pick-up/release applications due to the temperature-sensitive P(OEGMA-DSDMA) layer.^267^ Because of the pH-responsive property of 2-hydroxyethyl methacrylate (PHEMA), the PHEMA/PEGDA-Fe_3_O_4_ bilayer soft microrobot formed via photolithography (Figure 11B) performed the trapping of drug microbeads at about pH 9.58 by full folding motion and the release of drugs by unfolding motion at about pH 2.6.^266^

Biocompatible magnetic “hairbots,” derived from functionalized hair (Figure 11C), can display heightened osteogenic differentiation capacities of mesenchymal stem cells under magnetic actuation compared with nonmagnetic hairbots. Moreover, a magnetic field with repulsion mode endowed stem cells with higher osteogenic activity compared with the attraction equilibrium or nonequilibrium mode.^268^ Liquid metals (LM) have also been recently used to create shape-morphing flexible microrobots. An ice-assisted transfer printing method was used to fabricate Fe_3_O_4_NPs-incorporated EGaIn LM micromotors (Figure 11D). Because ice can be easily removed, this method provides great convenience for transferring LM-based micromotors to arbitrary desired substrates. Irradiation from an alternating magnetic field could cause the dramatic morphological transformation of LM-based micromotors in an aqueous environment. Moreover, the resulting LM-based microswimmer exhibited high propulsion velocity (over 60 μm s^–1^) under an elliptically polarized magnetic field as compared with its rigid counterparts.^269^

The utilization of DNA as a flexible component is another method to create soft micro/nanorobots is shown in Figure 11E. Artificial flagella with a length of several micrometers were generated using a self-assembled DNA bundle.^270^ After attaching the soft DNA flagella to a magnetic microbead via biotin–streptavidin coupling interaction, a hybrid microrobot was constructed. The fabricated magnetic microrobots can be propelled like peritrichous bacteria under a homogeneous rotating magnetic field. Similarly, Rémi Dreyfus and co-workers^159^ used biotinylated double-stranded DNA as “soft” hinges to link red blood cells decorated with streptavidin-modified superparamagnetic particles. In this way, another type of flexible artificial flagella was prepared via the specific biotin–streptavidin interaction.

Origami as a self-folding process provides a top–down approach to fabricate soft robots with transformable morphologies. A complete origami robotic system normally comprises power, sensing, actuation, and computation subcomponents.^271−274^ Readers are suggested to read the review article written by Daniela Rus and Michael T. Tolley to obtain more information about the design, fabrication, and control of origami robots.^275^ Self-folding origami MagRobots with various body designs (i.e., tubular body and helical tail, tubular body and spiral tail, helical body and planar tail, etc.) were created by Nelson’s group.^60,63^ The micro-origami swimmers were endowed with reconfigurable morphologies, controllable mobility, and even programmable magnetic anisotropy by embedding magnetic nanoparticles into self-folding hydrogel bilayers (i.e., one supporting layer and one thermally responsive layer). Because of the programmable shape-morphing feature of the origami-based microrobots, an artificial microsized “bird” was created to mimic the different flying modes of a real bird, including “flapping,” “hovering”, “turning”, and “side-slipping” (Figure 11F).^64^

### 4.4 Wire-like MagRobots

**Figure 12:**
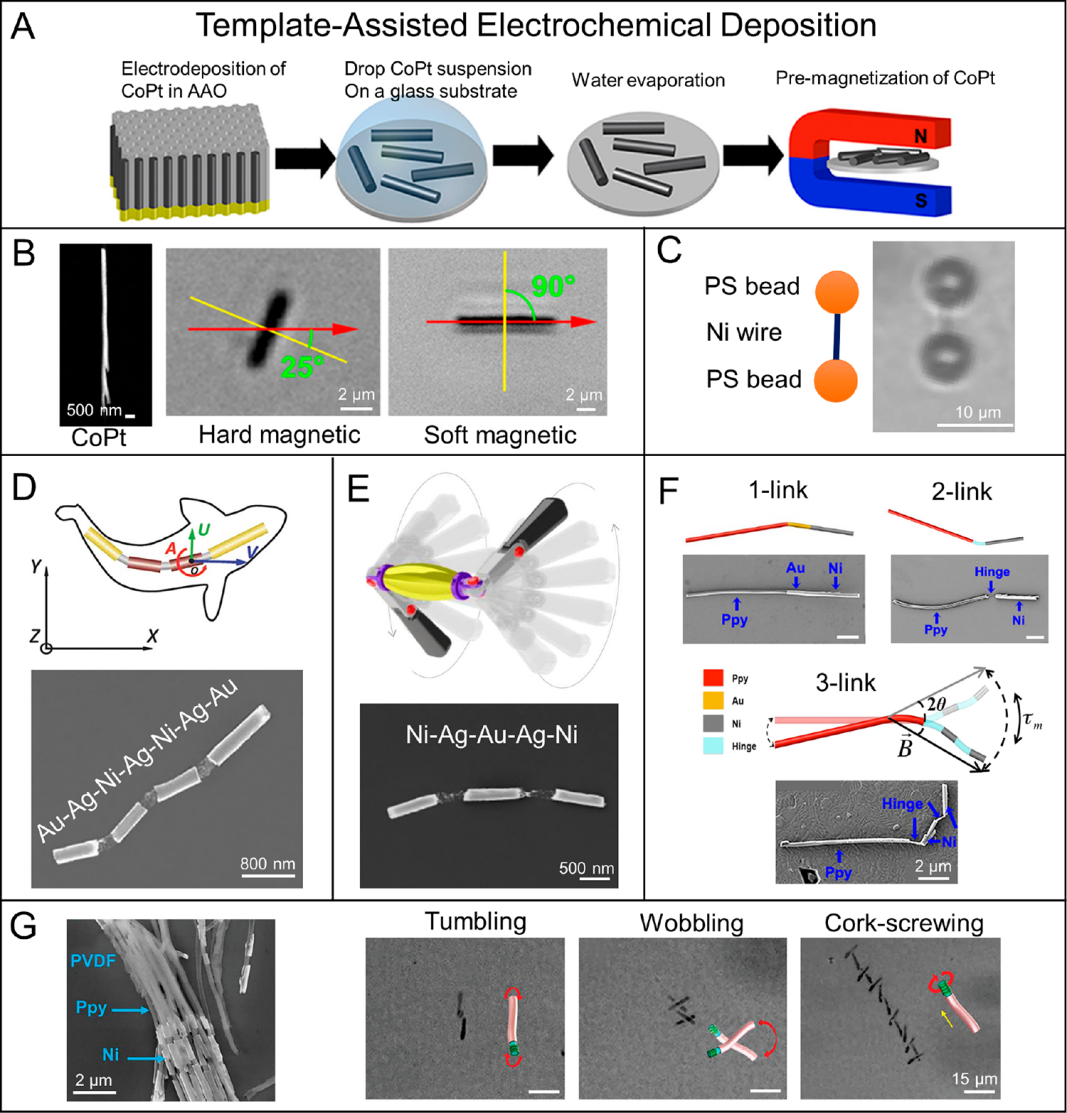
Fabrication of magnetic nanowires by TAED and some examples. (A) Synthesis process of CoPt nanowires and (B) magnetization angle of hard-magnetic CoPt nanowire and soft-magnetic CoNi nanowire. Yellow indicates the direction of the short axis while red indicates the direction of the magnetic field. Reproduced with permission from ref (166). Copyright 2019 American Chemical Society. (C) Dumbbell-shaped MagRobot consisting of a Ni NW and two PS microbeads. Reproduced with permission from ref (286). Copyright 2016 WILEY-VCH Verlag GmbH and Co. KGaA, Weinheim. (D) Traveling-wave motion of a fish-like nanoswimmer under an oscillating magnetic field. Reproduced with permission from ref (129). Copyright 2016 WILEY-VCH Verlag GmbH and Co. KGaA, Weinheim. (E) Freestyle swimming of two-arm nanoswimmer. Reproduced with permission from ref (3). Copyright 2017 American Chemical Society. (F) SEM images of 1-, 2-, and 3-link microswimmers and traveling-wave propulsion of 3-link microswimmer under an oscillating magnetic field. Reproduced with permission from ref (97). Copyright 2015 American Chemical Society. (G) Three motion modes and SEM image of PVDF-Ppy-Ni nanoeels. Reproduced with permission from ref (287). Copyright 2019 WILEY-VCH Verlag GmbH and Co. KGaA, Weinheim.

Most rod-like MagRobots are fabricated by template-assisted electrochemical deposition (TAED).^276−280^ In general, anodic aluminum oxide (AAO) or polycarbonate porous membranes are employed as templates. These membranes are commercially available and are usually composed of cylindrical pores, although sophisticated designs and complicated fabrication of porous membranes with different pore geometries or with variable pore diameter can be realized.^281,282^ Because of the nonconductive nature of these templates, prior to the electrodeposition of material, a layer of a conductive thin film (usually gold) is deposited on one side of the membranes by electron beam evaporation or other physical vapor deposition methods. The length of the nanostructures (i.e., nanorods, nanowires) is adjustable by regulating the electrodeposition time. After deposition, metal-based nanowires are released by dissolving the membrane template. Usually, ferro- and ferrimagnetic nanowires and nanorods align with their long axis parallel with the direction of the applied magnetic fields. Two main strategies exist to align cylindrical magnetic nanostructures perpendicular to their long axis: (a) by placing segments of magnetic material sufficiently separated along a nonmagnetic structure (in order to minimize dipolar interactions) and (b) premagnetizing the nanowires/nanorods along their short axis. The first case can be achieved by synthesizing multisegmented nanowires/nanorods using pulsed plating electrodeposition or sequential deposition by alternating different electrolytes.^283,284^ In the second approach, a nanowire/nanorod has to be made from hard-magnetic materials so that it can preserve a sufficiently large remanence after being premagnetizing in a specific direction. Figure 12A shows the fabrication of electrodeposited hard-magnetic CoPt nanowires and the procedure for their premagnetization along their short axis.^166^Figure 12B shows a comparison between a soft-magnetic CoNi and a hard-magnetic CoPt nanowire and their alignment upon the application of a magnetic field. While the premagnetized hard-magnetic nanowire aligns with its short axis to the applied field, the soft-magnetic is aligned along its long axis. In a rotational magnetic field, a nanowire/nanorod that aligns with its long axis with the applied magnetic field can only exhibit a tumbling motion.^285^ However, a nanowire-like MagRobot that is premagnetized along its short axis can display a richer variety of motion mechanisms such as tumbling, rolling, precession, or wobbling locomotion as a function of the magnetic field frequency. Another strategy to possess multiple motion modes is to integrate premagnetized nanowires into nonmagnetic structures. For instance, a single Ni nanowire only shows a sole tumbling motion.^285^ After assembling two polystyrene beads into a Ni nanowire to construct a dumbbell-like MagRobot, the fabricated microstructure possesses three motion modes (i.e., rolling, wobbling, and tumbling) (Figure 12C).^286^

When adding flexible segments such as hinges or tails to nanowires, the assembled MagRobots display traveling-wave motion under the steering of an oscillating magnetic field. A multiple section microstructure of Au–Ag–Ni–Ag–Ni–Ag–Au, using three elastic Ag nanowires as hinges and fabricated by sequential electrochemical deposition, can mimic the swimming of a fish with a speed as high as 30 μm s^–1^ (Figure 12D).^129^ In a similar fashion, the two arms of a Ni–Ag–Au–Ag–Ni MagRobot are capable of executing an out-of-phase wobbling motion by a planar 2D oscillating field and propel the movement of the body with a velocity of around 30 μm s^–1^ (Figure 12E).^3^ A Ni-hinge-Ni-hinge-Ppy nanorobot involving a flexible polypyrrole (Ppy) tail has the ability to break the reciprocal motion at the temporal dimension, exhibiting an S-like motion mode by making use of its eukaryote-like tail with the assistance of an oscillating field, leading to maximum propulsion speed of 0.93 body-lengths s^–1^ (Figure 12F).^97^

Inspired by the electric field, a knifefish, which can produce electricity through its electrocytes, was developed as a multifunctional Ni-Ppy-PVDF MagRobot containing a soft polyvinylidene fluoride (PVDF) tail. Taking advantage of the intrinsic piezoelectric performance of the PVDF tail, the surface of the fabricated MagRobots exhibits an enhanced release of cargo owing to the electrostatic repulsion generated by the magnetically induced piezoelectric effect. By changing the magnitude and rotational frequencies of the applied rotating magnetic field, three different locomotion modes (i.e., tumbling, wobbling, and corkscrew-like motion) with different translation speeds and drug release behaviors were observed (Figure 12G). Interestingly, the application of an on–off magnetic field can actuate the release of drugs in a pulsatile approach.^287^

### 4.5 Biohybrid MagRobots

**Figure 13:**
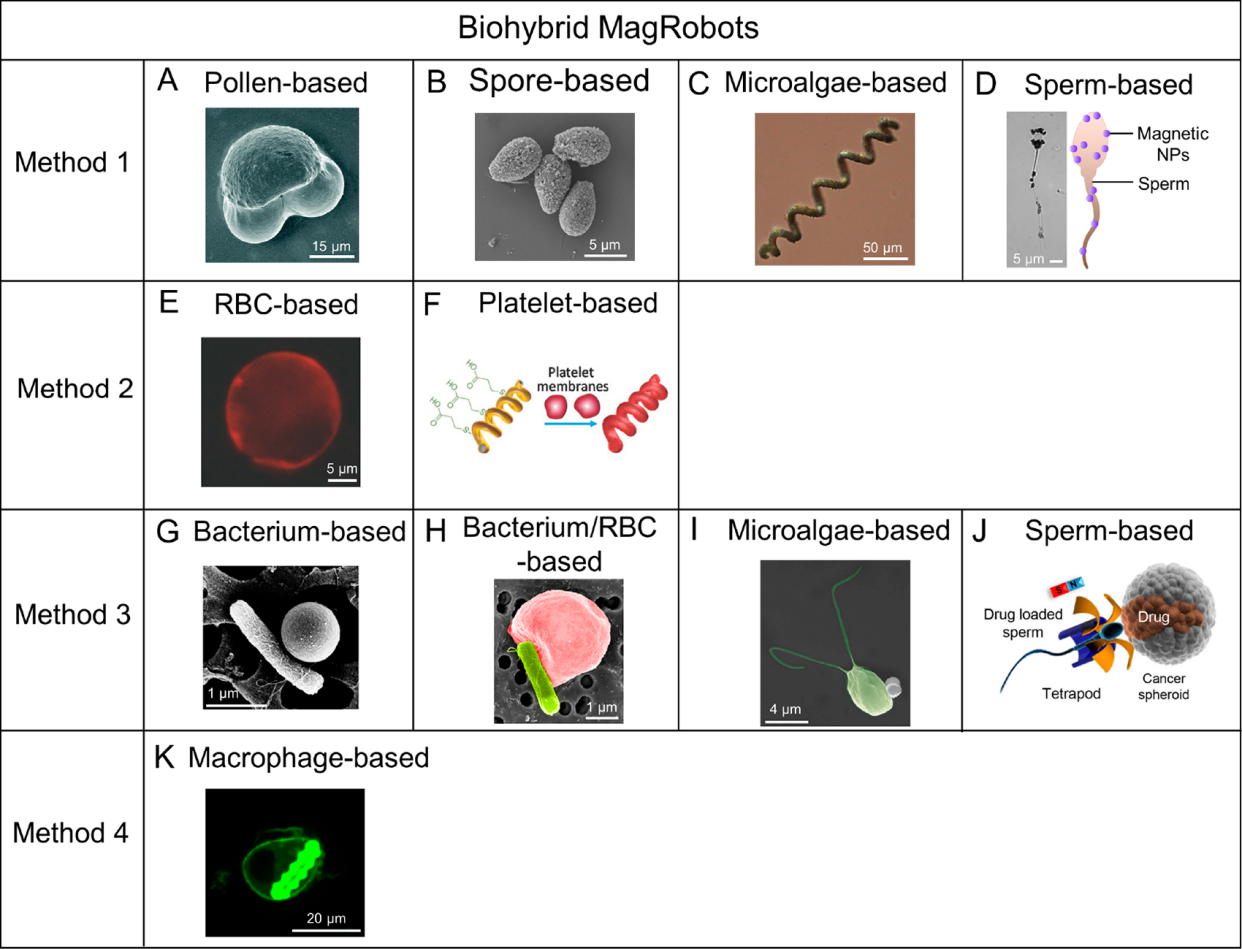
Representative examples of biohybrid MagRobots fabricated by four methods. Method 1: MagRobots prepared using (A) pollen, (B) spore, (C) microalgae, or (D) sperm as templates. Method 2: MagRobots prepared by cloaking functionalized nanomaterials with cell membrane of (E) red blood cells or (F) platelets. Method 3: MagRobots prepared by combining active flagella-containing cells such as (G) bacterium, (H) RGB-cloaked bacterium, (I) microalgae, or (J) sperm. Method 4: MagRobots prepared by utilizing the phagocytosis function of immune cells, for example, (K) macrophage. (A) Reproduced with permission from ref (289). Copyright 2019 The Royal Society of Chemistry. (B) Reproduced with permission from ref (296). Copyright 2019 The Authors, some rights reserved; exclusive licensee American Association for the Advancement of Science. (C) Reproduced with permission from ref (293). Copyright 2019 American Chemical Society. (D) Reproduced with permission from ref (295). Copyright 2020 The Authors, some rights reserved; exclusive licensee American Association for the Advancement of Science. (E) Reproduced with permission from ref (297). Copyright 2015 WILEY-VCH Verlag GmbH and Co. KGaA, Weinheim. (F) Reproduced with permission from ref (216). Copyright 2017 WILEY-VCH Verlag GmbH and Co. KGaA, Weinheim. (G) Reproduced with permission from ref (68). Copyright 2017 American Chemical Society. (H) Reproduced with permission from ref (306). Copyright 2018 The Authors, some rights reserved; exclusive licensee American Association for the Advancement of Science. (I) Reproduced with permission from ref (294). Copyright 2018 WILEY-VCH Verlag GmbH and Co. KGaA, Weinheim. (J) Reproduced with permission from ref (31). Copyright 2018 American Chemical Society. (K) Reproduced with permission from ref (305). Copyright 2020 The Authors, some rights reserved; exclusive licensee American Association for the Advancement of Science.

Because of their excellent biocompatibility and extremely low toxicity, biohybrid mineralized motors, which often integrate synthetic nanostructures/nanoparticles with natural nonmobile cells (e.g., pollen, spores) or motile cells (e.g., bacteria, sperm), are currently of great interest.^136,288^ Four methods are commonly used to produce biohybrid micro/nanorobots. The first method consists of directly using nonmotile cells as templates and then integrating magnetic nanomaterials and other functional building blocks such as inorganic nanostructures or molecules. Capitalizing on this approach, several pollen-based,^289−291^ spore-based,^292^ microalgae-based,^293,294^ sperm-based^295^ magnetic micromotors have been fabricated. In general, pollen and spores have the merits of excellent biocompatibility characteristics and structural uniformity. Some even have unique architecture (e.g., hollow cavity), which can facilitate specific applications. For instance, researchers have loaded drugs into two hollow air sacs of pine pollen grains via vacuum loading technique (Figure 13A). The experiments demonstrated that pollen-based biohybrid MagRobots not only exhibit efficient drug-encapsulation ability but also can release them on demand.^289^ By altering the vectors of programmatically controllable magnetic fields, individual pollen-based micromotors with encapsulated magnetic Fe_3_O_4_ inside present three distinct modes of locomotion (i.e., rolling, tumbling, and spinning) and these individuals were able to form a dynamic collective phenomenon under the steering of an external magnetic field.^289^ Spore-based microrobots composed of *G. lucidum* spores, Fe_3_O_4_ nanoparticles, and functionalized carbon nanodots have been synthesized via rapid, direct, and low-cost methods (Figure 13B). The prepared spore@Fe_3_O_4_@CDs microrobots can detect bacterial toxins.^296^ As mentioned above, Spirulina, with the innate spiral morphology, has been utilized as a biological template to create helical microswimmers^249,293^ (Figure 13C). Sperm-based soft MagRobots were fabricated by decorating Fe_2_O_3_ nanoparticles on the surface of immobile sperm cells via the electrostatic self-assembly (Figure 13D). The highest swimming speed of sperm-templated micromotors can reach 6.8 ± 4.1 μm s^–1^ (0.2 body length/s).^295^

The second method of preparing biohybrid micromotors is to cloak functionalized synthetic nanomaterials with cell membranes. This method can enhance the biocompatibility of micromotors to the largest extent and avoids recognition by the immune system. Recently, cell membranes/vesicles from red blood cells (RBCs)^297^ (Figure 13E), platelets^216^ (Figure 13F), and even dual cells (e.g., RBCs and platelets^298^) were utilized as camouflage to cover the surface of functionalized synthetic nanomaterials. The magnetic nanoparticles embedded into these biohybrid nanomachines play a role in magnetic guidance. The locomotion of these cell-based biohybrids can be powered by a magnetic field or other driving forces. For example, the random movement pattern of a Janus RBC-Mg motor can be driven by hydrogen bubbles generated by the reaction of Mg and water. The addition of Fe_3_O_4_ nanoparticles to the Janus micromotors can make the miniaturized machines move precisely along a predetermined path.^297^

The third method to fabricate hybrid small-scale swimmers consists of combining active locomotive cells that are born with flagella, among which sperm and bacteria are widely used.^31,68,299−302^ In this method, the motile cell either adheres to the surface of a synthetic particle (normally in the micrometer scale) or another cell or be trapped into a special microstructure. For example, bacteria-driven microswimmers were fabricated by attaching a single *E. coli*. bacterium to a drug-loaded polyelectrolyte microparticle via viscoelastic connection of the bacteria–particle interface (Figure 13G). The *E. coli*-powered motor exhibited the chemotaxis behavior under a chemical concentration gradient. Fe_3_O_4_ nanoparticles embedded within the polyelectrolyte microparticles functioned as a steering wheel, thus providing the biohybrid motors with directional control over the directionality and enabling guidance of the drug-loaded swimmers to target breast cancer cells *in vitro*.^68^ Similarly, the magnetic guidance was also employed in bacterium-RBC micromotors, which were fabricated through the strong conjugation chemistry between the erythrocyte and *E. coli* bacterium (Figure 13H). In addition, negatively charged microalgae with ellipsoidal morphologies (i.e., *Chlamydomonas reinhardtii algal*) were integrated with positively charged polyelectrolyte-functionalized magnetic microsphere via electrostatic interactions (Figure 13I). The motile microalgae function as an actuator while the microparticle can be used for cargo encapsulation and magnetic steering.^294^ In addition, various customized magnetic microstructures (such as tetrapod,^31^ microtube,^299^ and helix^248^) have been prepared to capture the task-carrying spermatozoa to form sperm-hybrid microrobots (known as “spermbots”). Sperm cells with high vitality serve as a motile component of hybrid microrobots to complete specific tasks, for example, targeted drug delivery,^31^ as shown in Figure 13J. However, they can also act as carriers when they have motility deficiencies. In such cases, the remotely controlled assisted fertilization relies on the synthetic magnetic microstructures of spermbots under the guidance of external magnetic fields.^303^

The fourth approach consists of adopting a live immune cell to engulf the whole magnetic passive functional materials by taking advantage of the phagocytosis processes of immune cells.^304^ As a consequence, biohybrid “immunobots”,^305^ as termed by Metin Sitti’s group, can be formed. After a magnetic double-helical microswimmer was completely internalized by a macrophage, the biohybrid macrophage-based MagRobots were able to perform magnetically driven rolling locomotion along predetermined trajectories by steering the magnetic helical component. The robots were able to swim uninterruptedly even with the presence of cells blocking their pathway. In the absence of a magnetic field, the immunobots could autonomously move by crawling and actuated by the self-propelled movement of the macrophages in a biological environment (Figure 13K).^305^

## 5 Applications

### 5.1 Targeted Drug/Gene Delivery

The precise and efficient transportation of therapeutic payloads to target sites, especially to those confined and hard-to-reach locations of the body, is challenging for passive drug delivery systems. The past decade has witnessed a boom in the development of active smart drug delivery systems using external field-driven miniaturized micro- and nanomotors. Particularly, magnetically driven micro and nanorobots offer several advantages as small agents for targeted cargo delivery including but not limited to remote, precise, and minimally invasive maneuverability, and potential recyclability of residual administered drug-carriers, which often results in serious side effects to healthy organs and tissues.^307−310^ In most cases, very low field strength (in the mT range) is sufficient for the actuation of MagRobots without causing damage to healthy cells.

**Figure 14:**
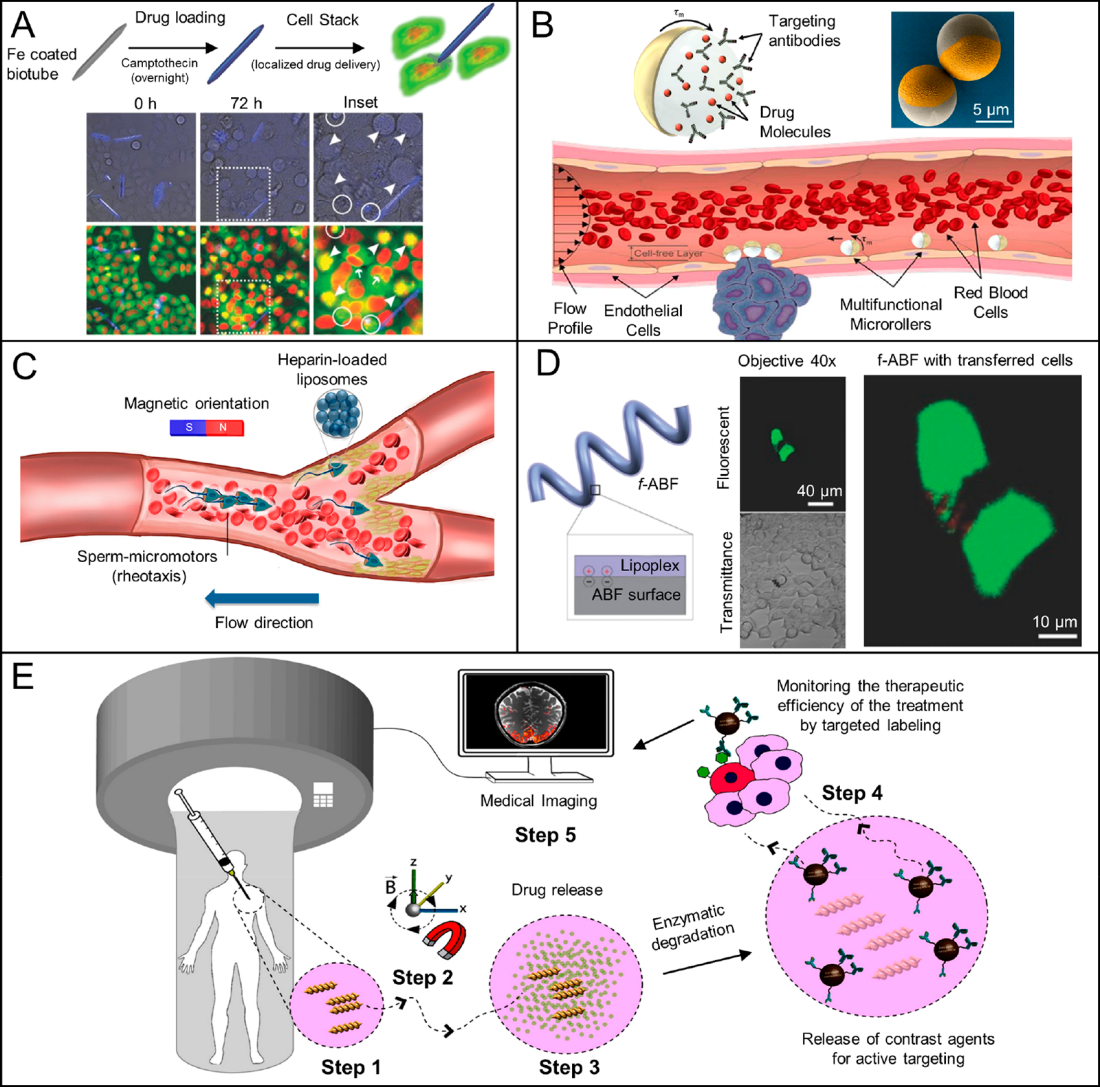
Magnetically powered micromotors for targeted cargo delivery. (A) Fe-coated camptothecin-loaded magnetic biotube for killing HeLa cells. Dead cells are highlighted by white circles. Reproduced with permission from ref (315). Copyright 2015 WILEY-VCH Verlag GmbH and Co. KGaA, Weinheim. (B) Controllable navigation and targeted transport of antibodies inside blood flow by using Janus micropropellers. Reproduced with permission from ref (312). Copyright 2020 The Authors, some rights reserved; exclusive licensee American Association for the Advancement of Science. (C) Sperm-based MagRobots capable of delivering heparin-loaded liposomes through flowing blood. Reproduced with permission from ref (177). Copyright 2020 American Chemical Society. (D) pDNA transfection by human embryo kidney cells when in targeted contact with helical microrobots loaded with plasmid DNA. Reproduced with permission from ref (219). Copyright 2015 WILEY-VCH Verlag GmbH and Co. KGaA, Weinheim. (E) Released drugs from hydrogel-based microswimmer for active labeling. Reproduced with permission from ref (128). Copyright 2019 American Chemical Society.

Before the steerable delivery of cargos (e.g., molecules, drugs, genes), the cargo loading or capture process is needed. The loading of cargos is often conducted by encapsulating them inside the MagRobot structure or by attaching them to the MagRobot surface. The encapsulation process can be directly carried out during MagRobot fabrication while the surface attachment (or adhesion) process can be made using superficial functional groups of biohybrid or synthetic MagRobots. Various organic or inorganic artificial nanomaterials (e.g., Au/Ni/Si nanospears,^311^ hydrogel-based helical microswimmers,^128^ Janus Au/Ni/SiO_2_ microparticles,^312^ etc.) and biogenic materials (such as pollen grains,^289^ sperm cells,^177^ bacteria,^35,306,313^ erythrocytes,^314^ and microalgae^247,294^) have been developed as functional or structural carriers to encapsulate or carry molecules, drugs, genes, or cells. For example, Fe-coated biotubes, which exhibit a drill-like motion under high-angular frequency magnetic fields, were capable of transporting camptothecin (i.e., an anticancer model drug) and delivering it to specific sites, killing the targeted HeLa cells *in vitro* (Figure 14A).^315^

Considering the complexity of the human body’s environments, it is key to investigate the propulsion mechanisms of MagRobots and strategies for cargo delivery and release under complicated physiological conditions in different body fluids such as gastric juice, saliva, and blood. Recently, a cell-sized Janus micromotor loaded with antibodies as receptors for the recognition of target cells and anticancer drugs was able to navigate in a simulated blood circulation system (Figure 14B).^312^ Although the propulsion of MagRobots was weakened under dynamic flow conditions, the ability of active upstream locomotion in the bloodstream was confirmed in flat and 3D surfaces. Furthermore, the utilization of biohybrid micromotors combining sperm cells and synthetic magnetic micro and nanoarchitectures to deliver anticoagulant agents (i.e., heparin) in the bloodstream was reported (Figure 14C),^177^ which is promising for treating diseases of the circulatory system such as thrombotic clots. In addition to drugs, targeted transport of genes (e.g., plasmid DNA) to a single cell and subsequent transfection was achieved by the utilization of helical micromotors under the actuation and navigation of low-strength rotating magnetic fields (Figure 14D).^219^ Recently, Peer Fischer’s group reported targeted transfection and gene delivery by using biocompatible FePt nanopropellers under rotating millitesla fields.^316^

After delivering payloads to a specific location, cargo molecules can be released naturally via diffusion or via specific stimuli (such as pH,^266^ temperature,^267^ light irradiation,^67^ or chemical changes at the disease site) according to the practical application requirement. For example, because the concentration of matrix metalloproteinase-2 (MMP-2) enzyme at the tumor site is higher than that at normal physiological conditions, hydrogel-based helical microswimmers demonstrate a quicker response to the evaluated concentration of MMP-2 enzyme, resulting in a boost-release of embedded cargo (i.e., antibody-tagged Fe_3_O_4_ nanoparticles) through the swell behavior of the hydrogel.^128^ The released antibody-tagged payloads from the micromotors can be further used for active labeling of targeted tumor cells (Figure 14E).

### 5.2 Cell Manipulation

**Figure 15:**
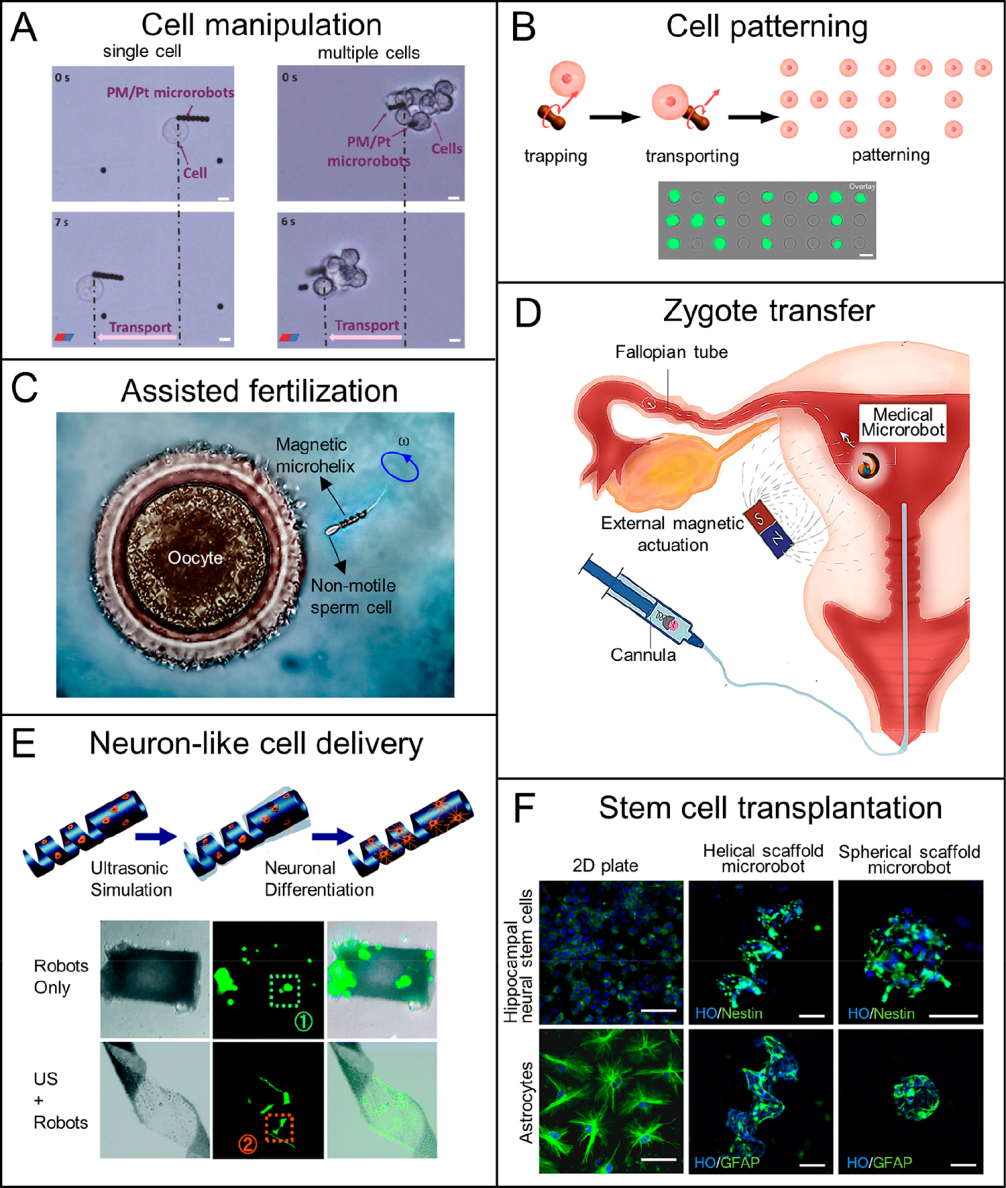
MagRobots for cell manipulation. (A) Manipulation of T47D cancer cells using superparamagnetic/Pt Janus micromotors via bubble propulsion and magnetic actuation. Reproduced with permission from ref (210). Copyright 2018 WILEY-VCH Verlag GmbH and Co. KGaA, Weinheim. (B) Delivery and patterning of a single cell by peanut-like hematite microrobots. Reproduced with permission from ref (170). Copyright 2018 American Chemical Society. (C) Transport of nonmotile sperm cells to the oocyte with the assistance of magnetically driven helical micromotors. Reproduced with permission from ref (248). Copyright 2015 American Chemical Society. (D) Magnetically powered microspirals for the delivery of murine zygote. Reproduced with permission from ref (321). Copyright 2020 The Authors. (E) Magnetically actuated transport of neural progenitor cell and ultrasound-induced neuronal differentiation. Reproduced with permission from ref (217). Copyright 2019 The Royal Society of Chemistry. (F) MagRobots as motile 3D scaffolds for stem cell delivery. Reproduced with permission from ref (122). Copyright 2019 The Authors, some rights reserved; exclusive licensee American Association for the Advancement of Science.

Cell manipulation is the practice of maneuvering the physical position of cells to separate them from the milieu of other phenotypically different cells (i.e., cell-based screen), guiding them into a specific target position (e.g., for fertilization), or organizing themselves *in vitro*. With the rapid advance of proteomics and genomics, it is of great significance to develop sophisticated tools for single-cell manipulation, especially massively parallel single-cell manipulation.^317^ Magnetically powered miniaturized robots are capable of 3D manipulation of a single cell in terms of capture, transport, sorting, isolation, and pattering, with excellent maneuverability and high precision at the nano- and microscale in complex physiological environments without changing the intrinsic properties of the cells.^318,319^ For instance, trapping of breast cancer cells was reported by tosyl-functionalized superparamagnetic microbeads due to the instantaneous strong binding between the tosyl groups from the surface of microswimmers and the −NH_2_ groups from the membrane proteins of cancer cells. Manipulation of single or multiple cell-laden microrobots was achieved by the propulsion of oxygen bubbles and manual direction guidance using a neodymium magnet (Figure 15A).^210^ Arranging cells to achieve predetermined patterns with the assistance of an arrayed substrate was implemented through single-cell pick-up and subsequent delivery using magnetically propelled peanut-like micromotors (Figure 15B).^170^ To aid sperm cells with defective locomotion features to complete their fertilization task, Oliver G. Schmidt’s group designed several motile nano/micromotors as assisted tools^303^ such as magnetic microcarriers with a cylindrical cavity and a helical body^320^ and a magnetic helix^248^ (Figure 15C). Moreover, magnetically driven micromotors provide an invasive way to transfer zygotes through the uterus and fallopian tube (Figure 15D), and magnetic microrobots with spiral shapes exhibit higher maneuverability in terms of capture and transfer of the zygotes between different physiological environments than those with helical shapes.^321^ Transportation of neural progenitor cells was conducted by the corkscrew-like motion of magnetically powered soft microswimmers containing piezoelectric polymer and CoFe_2_O_4_ magnetic nanoparticles under a rotating magnetic field. Subsequent neuronal differentiation of PC12 cells was induced by the acoustic stimulation due to the utilization of piezoelectric polymer as a stimuli-responsive cell electrostimulation platform (Figure 15E).^217^ Furthermore, Kim et al.^322^ precisely manipulated a neuron-loaded magnetic microrobot to a gap between two neural clusters to connect broken neural networks. Recently, successful trials of magnetically powering microrobots toward a target site (such as a liver tumor micro-organ, ventricle of mouse brain, blood vessel of rat brain, and live mouse) using *in vitro*, *ex vivo*, and *in vivo* experimental models, indicate the feasibility of adopting MagRobots for the purpose of targeted stem cell transport and transplantation (Figure 15F).^122^

### 5.3 Minimally Invasive Surgery

**Figure 16:**
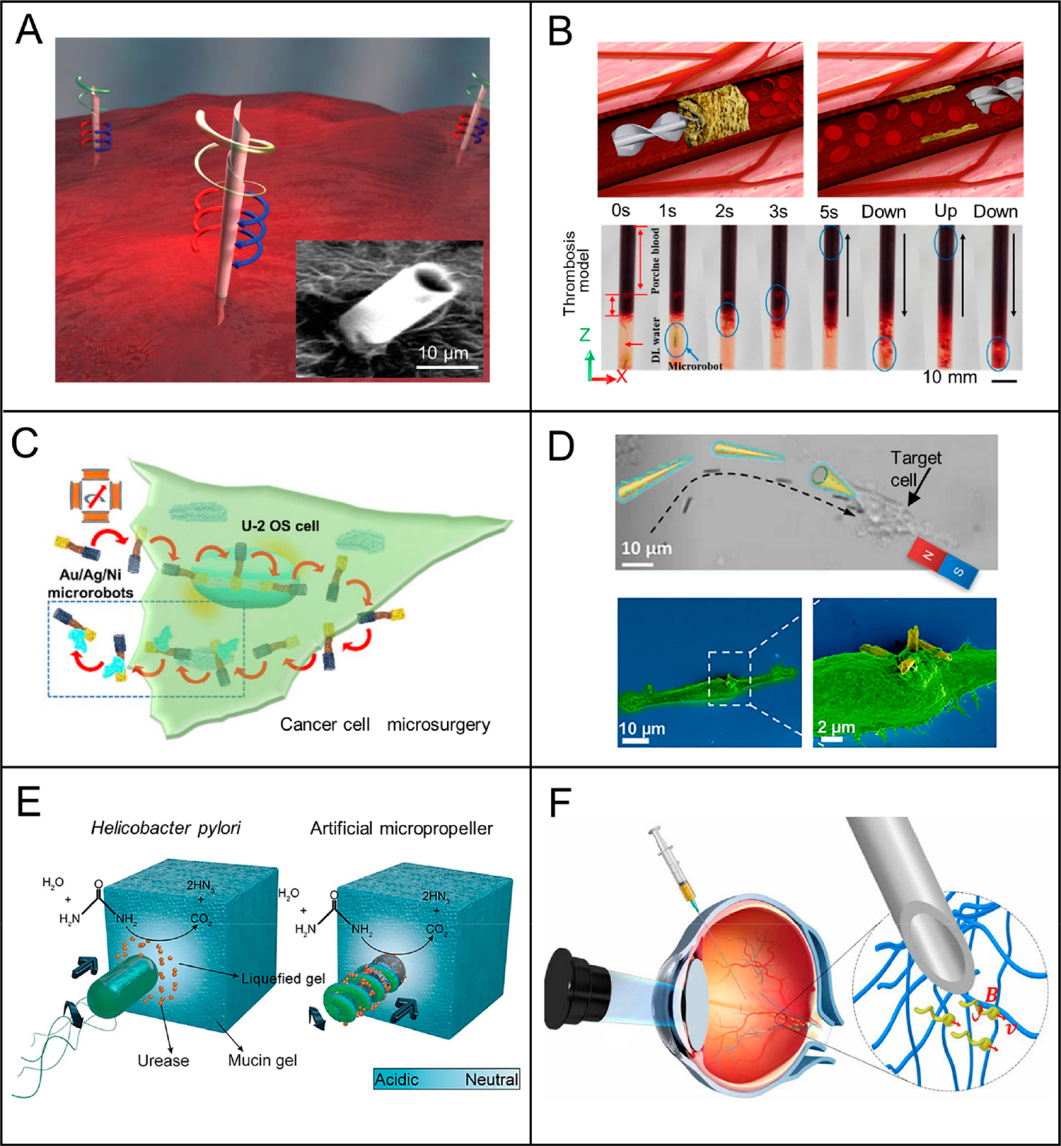
MagRobots for minimally invasive surgery. (A) Schematic image and experimental image (inset) of rolled-up magnetic microdrillers with sharp end penetrating into a pig liver after drilling motion. Reproduced with permission from ref (41). Copyright 2013 The Royal Society of Chemistry. (B) Schematic of a driller working in a 3D vascular network and experiment result shows the driller can dislodge blood clot. Reproduced with permission from ref (325). Copyright 2018 The Authors. This article is licensed under a Creative Commons Attribution 4.0 International License. (C) Movement of Au/Ag/Ni surface walker under a transversal rotating field with different frequencies and magnetic navigation of microrobots to penetrate a cell and remove a cell fragment. Reproduced with permission from ref (173). Copyright 2020 American Chemical Society. (D) Magnetic manipulation of Si/Ni/Au nanospears for targeted intracellular transfection. Reproduced with permission from ref (311). Copyright 2018 American Chemical Society. (E) Penetration of *Helicobacter pylori* bacterium and helical MagRobot into mucin gels and liquefaction of mucus via enzyme-catalyzed reaction. Reproduced with permission from ref (222). Copyright 2015 The Authors, some rights reserved; exclusive licensee American Association for the Advancement of Science. (F) Long-range propulsion of injected slippery MagRobots in the vitreous toward the retina with the assistance of a magnetic field and standard optical coherence tomography. Reproduced with permission from ref (127). Copyright 2018 The Authors, some rights reserved; exclusive licensee American Association for the Advancement of Science.

Miniaturized machines that are capable of precisely opening specific cell membranes to kill abnormal cells and even achieve intracellular delivery of various drugs (including DNA) are promising candidates for noninvasive surgery.^323,324^ Nano/microrobots that project sharp tips or have the ability to perform a corkscrew-like movement can execute drilling under the application of a rotating magnetic field. The drilling feature can be harnessed to penetrate tissue with high precision, holding great promise to perform untethered microsurgeries. As shown in Figure 16A, microdrillers (tubular Ti/Cr/Fe microdrillers with sharp tips) were able to penetrate into a section of porcine liver tissue via magnetically driven mechanical drilling. To make the microdriller “stand up” to drill, a specific angular frequency threshold of the rotating field (in correlation with the viscosity of media) is required to transform the horizontal rotation mode into a vertical rotation mode.^41^ Other representative microdrillers are Fe-coated calcified biotubes containing pointed ends, which are extracted from *Dracaenea marginata* leafs. Upon magnetic actuation, the microdagger stabbed into the cellular membranes of HeLa cells with a drill-like motion, finally resulting in cell death. In addition, the ability to drill into a target cell can be utilized for subsequent drug delivery because the porous structures of calcified biotubes endow the microdriller with the capacity of drug loading.^315^

A millimeter-sized magnetic driller can be navigated in a 3D vascular channel and perforate a blood clot in a simulated thrombosis model environment, providing an application potential for cardiovascular disorders (Figure 16B).^325^ Besides, surface walkers also can open the cell membrane. Recently, we developed Au/Ag/Ni microwires that display walking movement under a transversal rotating magnetic field. Because of the rigidness of the microwires, they can only perform a drilling movement. To make the structure of microwires slightly bent, an Ag segment was partly etched by concentrated H_2_O_2_ solution. As a consequence, a surface tumbling motion can be achieved. The surface walkers, functioning as microscalpels, can penetrate cancer cells, capture a piece of the cytosol, and exit the cells while leaving the cytoplasmic membrane intact, thus demonstrating excellent minimally invasive microsurgery capabilities^173^ (Figure 16C). Au/Ni/Si nanospears functionalized with plasmid were able to penetrate U87 glioblastoma cells by means of rotating magnets, and deliver the gene (i.e., eGFP expression-plasmid) within the cells over large areas (Figure 16D). Such intracellular cargo delivery in a high-throughput manner paves the way for translation to new clinical cellular therapies.^97,311^

Realistic biological environments are substantially complex. The microscopic propulsion of micro/nanorobots in biofluid environments (e.g., bloodstream,^24,251,326−328^ saliva,^329^ semen,^330^ mucus,^222^ vitreous humor,^127,331−333^ brain vasculature,^334^ cerebrospinal fluid in the spine or brain, urinary fluid, gastrointestinal fluid,^335,336^ etc.) is different from that in Newtonian fluid. Physicochemical and histological barriers (e.g., cell membrane,^323^ blood–brain barrier,^337^ intestinal mucosal barrier), interactions with boundaries, crowded biological environments, complex rheology (e.g., viscoelasticity, shear-thinning), and other factors impact the locomotion behaviors and application performance of micro/nanorobots in biological environments.^338−341^ Attempts have been made to exploit the actuation of MagRobots in complex biofluids. For example, to overcome the mucus barrier, Peer Fischer’s group^222^ developed a helical microdriller surface-functionalized with urease as shown in Figure 16E. Such microdrillers can penetrate the viscoelastic mucin gel in an acidic environment in the presence of urea and swim freely inside under a rotating magnetic field. This idea is inspired by *Helicobacter pylori* bacteria, which are capable of decreasing the viscosity of mucin gel via a gel–sol transition caused by the release of ammonia through an enzyme-catalyzed procedure that raises the local pH. To move further toward clinical application, the same group created magnetic helical micropropellers that were able to penetrate the biopolymeric network of porcine vitreous humor and swim inside over a centimeter distance under navigation by a rotating magnetic field and using clinical optical coherence tomography as shown in Figure 16F.^127^ The smooth propulsion of the micropropellers in the dense biopolymeric network lies in the slippery liquid layer on the surface of micropropeller, which minimizes the adhesion force to the surrounding environment. More mechanisms, actuation approaches, and applications of micro/nanorobots in complex biofluids that resemble real-world scenarios are required to be explored.

### 5.4 Biopsy

**Figure 17:**
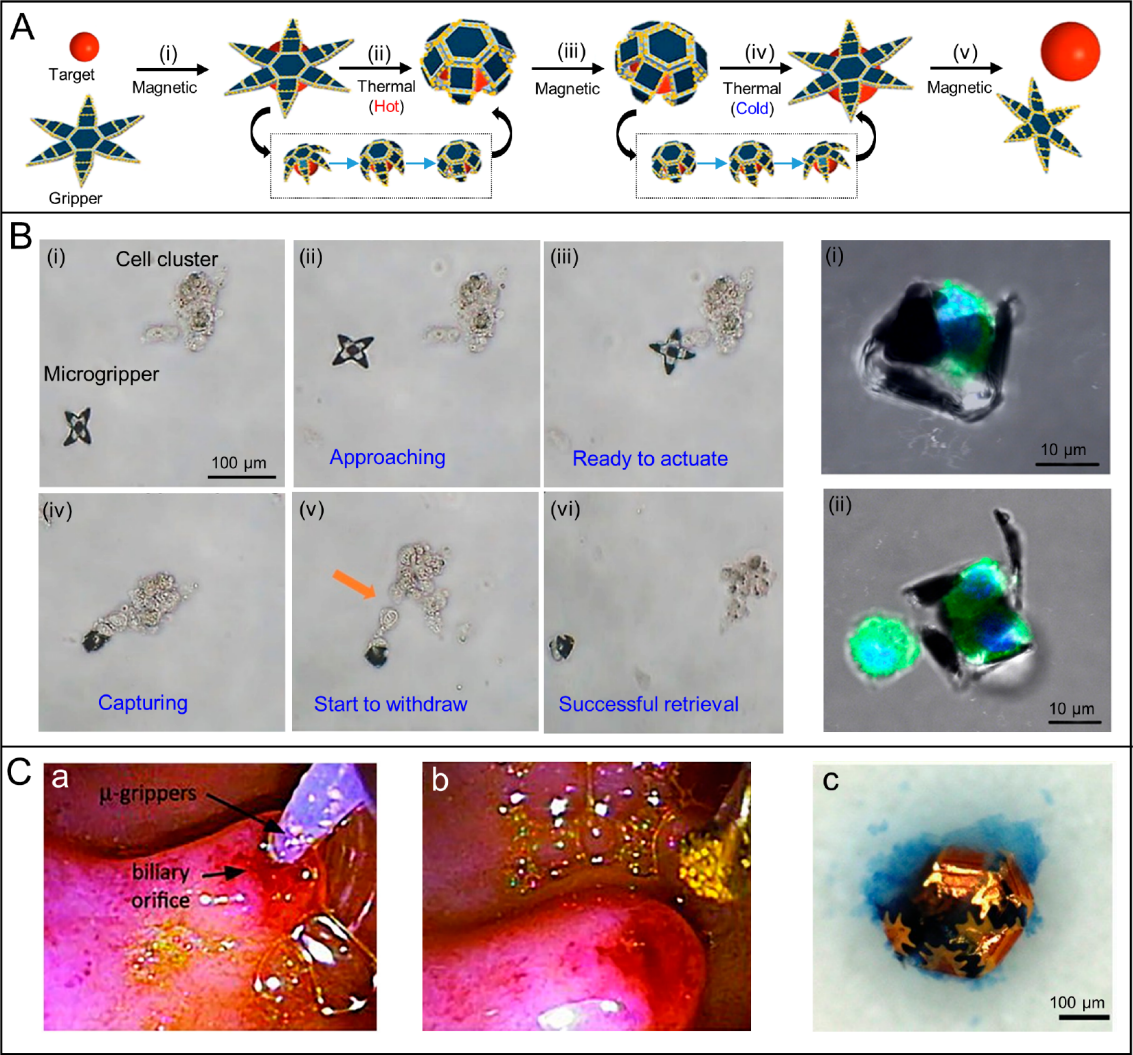
MagRobots for biopsy. (A) Schematic of a thermoresponsive gripper autonomously picking up and placing a target. Reproduced with permission from ref (345). Copyright 2016 The Authors. (B) Cell biopsy from a cell cluster using a magnetically navigated thermoresponsive microgripper and immunofluorescence images of suspended fibroblast cells captured by the microgripper. Reproduced with permission from ref (342). Copyright 2020 American Chemical Society. (C) (a) Transport of microgrippers into the porcine biliary orifice using an endoscope-assisted catheter; (b) retrieval of microrobots with the assistance of a magnetic catheter; (c) retrieved microrobot with a tissue piece in its “hand” after Trypan Blue staining. Reproduced with permission from ref (348). Copyright 2013 WILEY-VCH Verlag GmbH and Co. KGaA, Weinheim.

MagRobots have been proved to be wireless biopsy tools to capture a single cell or collect tissue samples from healthy or diseased organs, including breast, lung, liver, skin, prostate, and so forth, with high specificity and selectivity for further disease diagnosis. These functional magnetic miniaturized robots, normally in the microscale, are called microgrippers. To have the ability to pick up an object and lay it down, analogous to the function of human hands, most of the magnetically driven microgrippers^40,342−344^ explored to date are flexible (see Section 4.3). Thermoresponsive flexible MagRobots have been widely used as grippers due to their temperature-induced opening and closing capacities^39,345−347^ (Figure 17A). For instance, a thermoresponsive magnetic microrobot, having a tip-to-tip size of 70 μm in its open state and 15 μm in its folding state, was able to conduct single-cell biopsy (Figure 17B). The thermally responsive layer of the microgripper is made from paraffin wax, whose phase-transition temperature is in close proximity to biological temperatures, including humans. After being navigated to the position of a fibroblast cluster, the untethered microgripper grasped one cell or a few cells when it transformed from open to closed state with the increase of field temperature. Cell separation from the cluster and retrieval of the microrobot can be easily fulfilled by adjusting the direction of magnetic field. Metin Sitti’s group^343^ utilized hundreds of thermosensitive microgrippers that had been pre-encapsulated in the chamber of a centimeter-scaled magnetically actuated capsule endoscope (MASCE), to grab stochastically tissue inside the stomach *ex vivo* for further analysis. Retrieval of distributed magnetic microgrippers was conducted by strong wet-adhesive force from the retrieval unit of MASCE. This multiscale robotic system provides a novel multiagent collaboration strategy not only for gastrointestinal capsule biopsy but also for other biopsy tasks in complex physiological structures and environments. An *in vivo* tissue excision of the porcine biliary tree was conducted using thermal-induced self-folding microgrippers as shown in Figure 17C. More than 1000 microgrippers were delivered to the position of interest (i.e., the biliary orifice) through a standard catheter with the assistance of the endoscopic camera. The thermosensitive magnetic microrobots, initially in the open state, spontaneously transformed into closed state in order to excise tissue samples when they are exposed to body temperature (37 °C) for 10 min. Retrieval was carried out by using a catheter containing a magnetic tip. Subsequent PCR (polymerase chain reaction) results indicated that the excised tissue piece was sufficient for genetic or epigenetic diagnosis in terms of quantity and quality.

### 5.5 Biofilm Disruption/Eradication

**Figure 18:**
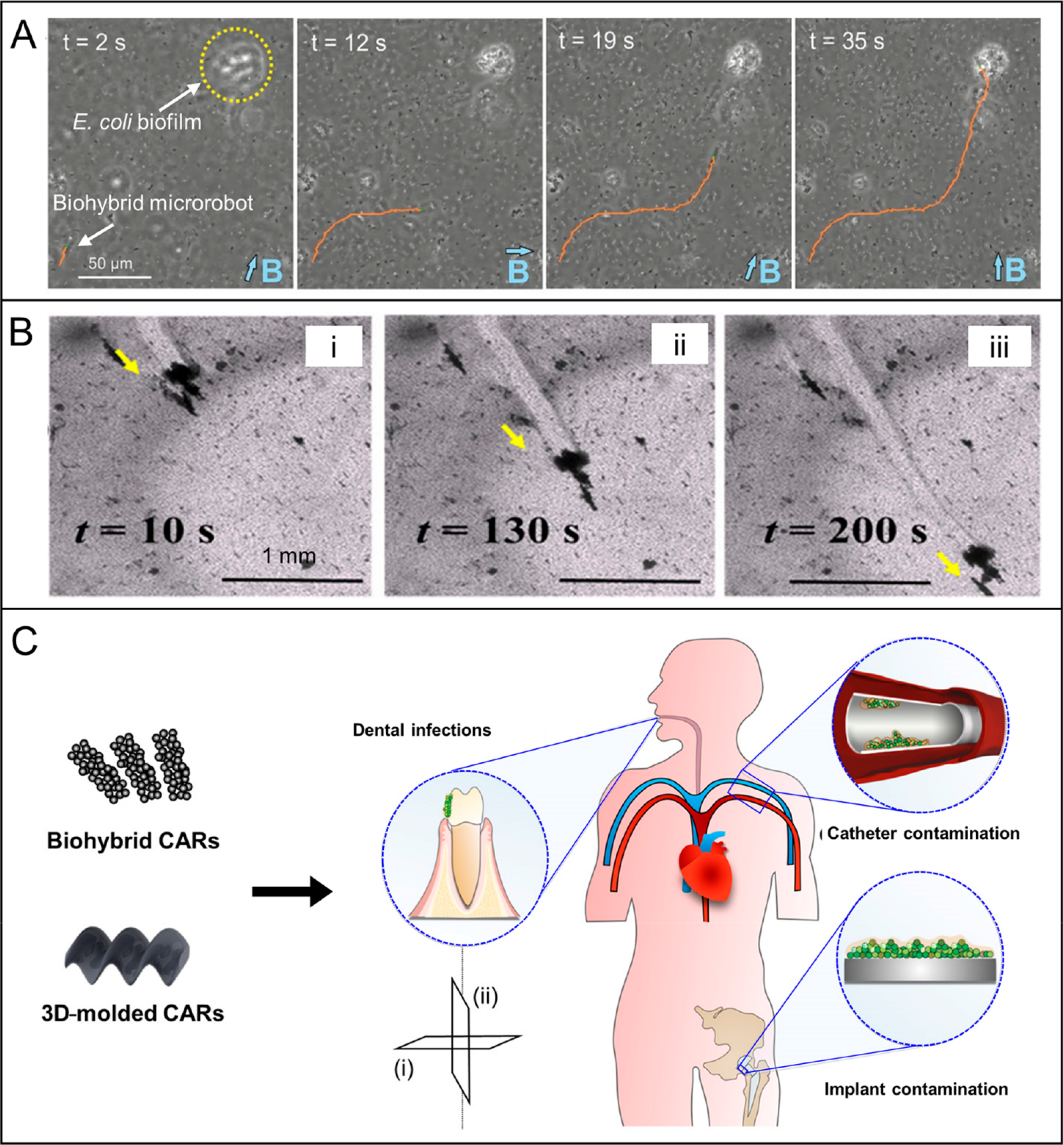
Representative examples of biofilm disruption or eradication using active MagRobots. (A) Magnetic guidance of biohybrid microbot into an island of *E. coli* biofilms. Reproduced with permission from ref (351). Copyright 2017 American Chemical Society. (B) Linear footprints left on the surface of *P. aeruginosa* biofilm after the motion of MagRobots. Reproduced with permission from ref (352). Copyright 2020 American Chemical Society. (C) Application illustration of biofilm removal in confined and hard-to-reach positions, such as interior of human teeth, catheter surfaces, or implant surfaces by using two types of catalytic antimicrobial robots (CARs) under the navigation of magnetic field. Reproduced with permission from ref (353). Copyright 2019 The Authors, some rights reserved; exclusive licensee American Association for the Advancement of Science.

Different from planktonic (free-swimming) bacterial cells, the interaction of cell masses (i.e., community of microorganisms) produces a matrix called “extracellular polymeric substances” (EPS).^349^ The embedded cells and the viscoelastic matrix that constitute the biofilm on the surface of a subject are notoriously difficult to eliminate.^350^ The nature of bacterial biofilms’ resistance to antimicrobial agents makes them a source of some recalcitrant infections. Magnetically powered nano/microrobots manifest themselves in the competence to penetrate into the matrix and disrupt the biofilm formation or eradicate already-formed biofilm due to their small size as well as high magnetically driven mechanical force. A biohybrid microrobot based on nonpathogenic magnetotactic bacteria has been used to penetrate into the island of *Escherichia coli* by the external actuation of magnetic field^351^ as shown in Figure 18A. Although this invasion can temporarily cause the elastic formation of the biofilm, the microrobot was almost trapped in it, presenting restrained movement ability. How to make nano/microrobots swim in a viscous media is a common challenge. A magnetic microrobot made from tea buds, called “T-Budbots”, was able to precisely fragment and remove bacteria biofilm.^352^ As demonstrated in Figure 18B, T-Budbots left a clear trail on the surface of *P. aeruginosa* biofilm after their movements, indicating that the biofilm had been effectively swept away. Moreover, antibiotic encapsulated in T-Budbots of the biofilm exhibited a pH-triggered release behavior around the acidic microenvironment of the biofilm. Once the biofilm was disrupted, the dislodged bacterial cells were exposed to the drugs and finally killed. One of the most outstanding advantages of using MagRobots to execute the task of biofilm elimination lies in their function to be directed to a confined and hard-to-access position. A recent study demonstrated that magneto-catalytic iron oxide nanorobots (called “CARs”) are capable of the degradation and removal of biofilms in the isthmus of human teeth due to the catalytically induced generation of reactive antibiofilm molecules and the external shear forces from magnetic actuation (Figure 18C).^353^

### 5.6 Imaging-Guided Delivery/Therapy/Surgery

To translate medical micro/nanorobots from the bench to the bedside, imaging technologies are of vital importance to achieve real-time tracking of the MagRobots *in vivo*.^202,354−361^ Clinically established imaging modalities, including but not limited to optical imaging, magnetic resonance imaging (MRI),^53,198,326,362−364^ magnetic particle imaging (MPI),^185^ fluorescence imaging,^365−367^ ultrasound (US) imaging,^89,125,368−371^ photoacoustic (PA) imaging,^226,372^ X-ray computed tomography (CT), photoacoustic computed tomography (PACT),^83^ optical coherence tomography (OCT),^127,373^ single-photoemission computed tomography (SPECT),^374^ positron emission tomography (PET),^375^ and their combined imaging techniques (e.g., MR/CT,^376^ PET/CT,^377^ PET/MRI^378^) can be integrated into miniaturized robotics systems. Although many challenges remain, many researchers have attempted to use these imaging techniques as powerful tools to assist the tracking of MagRobots for site-specific drug delivery, targeted therapy, and precision surgery.

**Figure 19:**
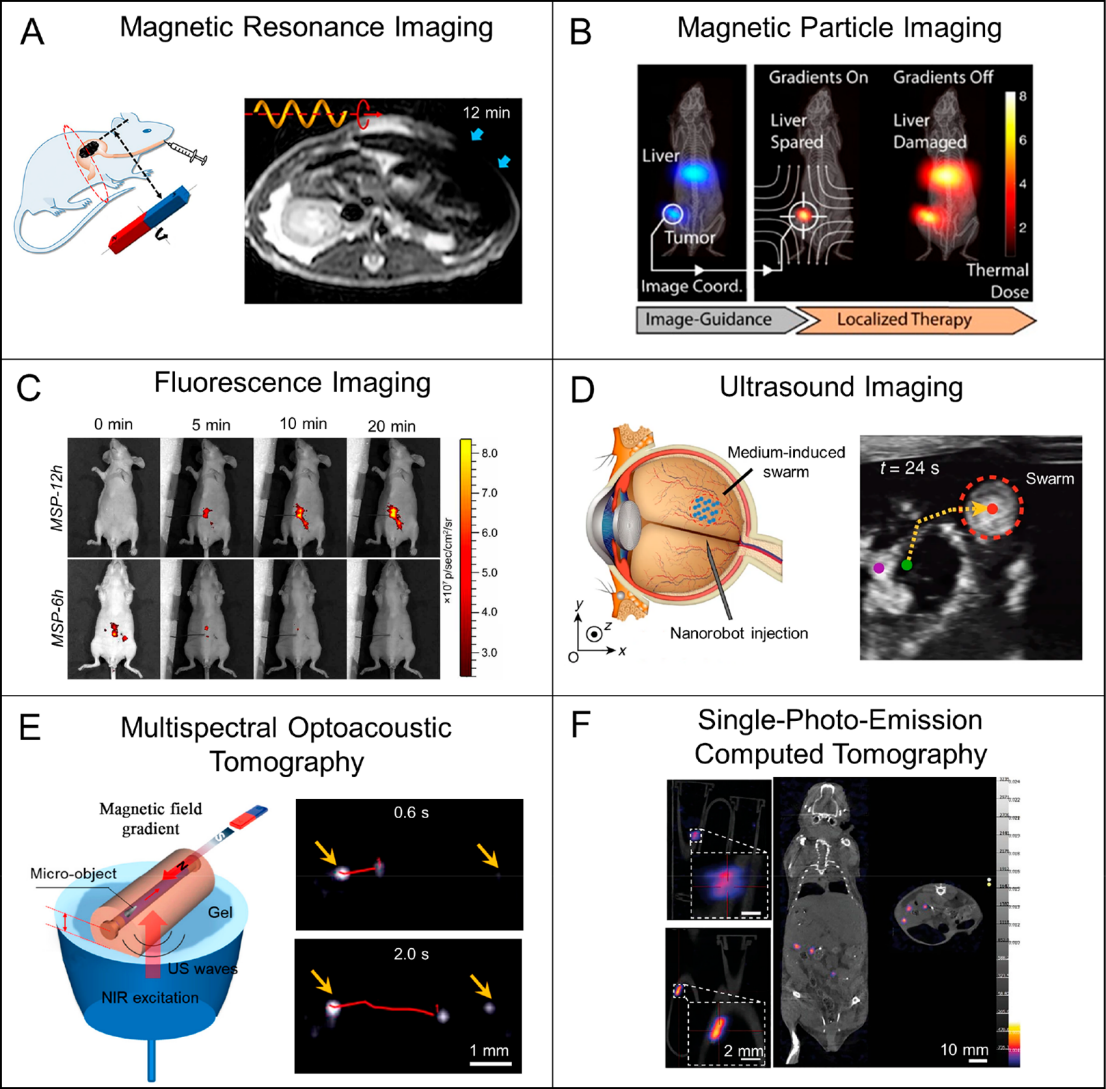
Visualization of MagRobots *in vivo via* various medical imaging modalities. (A) Cross-sectional magnetic resonance imaging of microrobot swarms inside the subcutaneous tissues of a rat’s stomach after magnetic actuation and steering via rotating field for different time periods. Reproduced with permission from ref (249). Copyright 2017 The Authors, some rights reserved; exclusive licensee American Association for the Advancement of Science. (B) Image-guided theranostic platform via the combination of magnetic particle imaging and localized magnetic hyperthermia experimentally demonstrated in a U87MG xenograft mouse with superparamagnetic nanorobots present in the liver and tumor. Reproduced with permission from ref (185). Copyright 2018 American Chemical Society. (C) *In vivo* fluorescence images of spirulina-based MagRobots in the intraperitoneal cavity of mice at various residence times. Reproduced with permission from ref (249). Copyright 2017 The Authors, some rights reserved; exclusive licensee American Association for the Advancement of Science. (D) Tracking of the generation process of a MagRobot swarm in a bovine eyeball via ultrasound imaging technique. Reproduced with permission from ref (125). Copyright 2019 The Authors. (E) Utilization of multispectral optoacoustic tomography for real-time tracking of individual moving microrobot within phantoms actuated by a permanent magnet. Reproduced with permission from ref (393). Copyright 2019 American Chemical Society. (F) SPECT images of radiolabeled microrobots in Eppendorf tube and in mice. Reproduced with permission from ref (374). Copyright 2019 WILEY-VCH Verlag GmbH and Co. KGaA, Weinheim.

Because of limited penetration depth of biological tissues, optical imaging is not suitable for the visualization of MagRobots across tissues *in vivo*. For magnetically driven micro/nanorobots, MRI is an efficient tool to track the position of MagRobots both *in vitro* and *in vivo*.^357^ Both MRI and MPI are magnetic-based imaging techniques. MRI has been widely used in clinical practice, especially for three-dimensional anatomical images of soft tissues. The main advantages of MRI lie in high soft-tissue contrast, high spatial resolution, and no consumption of dedicated contrast or imaging agents. Most importantly, strong magnetic fields and field gradients generated by MRI scanners provide suitable actuation environments for the navigation of MagRobots, while MagRobots with integrated magnetic compositions or components can augment the signal and boost image quality. As one representative example shown in Figure 19A, *in vivo* MRI tracking of a swarm of microalgae-based helical microrobot inside the subcutaneous tissues of a rodent stomach was reported by Zhang’s group.^249^ Felfoul and co-workers^379^ reported real-time positioning and tracking of a microrobots magnetically propelled by MRI gradients in the carotid artery of a pig in a closed-loop control scheme.

MPI, first proposed by Bernhard Gleich and Jürgen Weizenecker,^380^ is a three-dimensional tomographic imaging method. The MPI scanner comprises two permanent magnets in a Maxwell configuration. Larger field gradients in the MPI scanner workspace provide a strong propulsion force to drive magnetic objects.^381−383^ However, in terms of the spatial resolution of MPI (a few millimeters) in the current platform, this technique is only applied to the visualization of swarming micro/nanorobots, not an individual one. Tay and co-workers^185^ reported quantitative guidance of MPI imaging, precise localization of magnetic hyperthermia, induced by the interaction between MPI gradient and superparamagnetic magnetic nanoparticles, to arbitrarily selected tumor sites. When the field-free region (FFR) of the MPI gradient was centered to the targeted tumor area, localized heat only killed the cancerous tissues while minimizing the collateral heat damage to nearby healthy tissues (Figure 19B).

Fluorescence imaging, with the advantages of excellent planar resolution (≈ 100 nm) and high sensitivity, has become another widely used medical imaging modality. Under the guidance of fluorescence imaging, the utilization of spore-based magnetic microrobots functionalized with carbon quantum dots for effective targeted delivery was demonstrated by Zhang’s group.^384^ They designed an automated control system that can help microrobots avoid obstacles and find the optimal path based on a particle swarm optimization algorithm with the assistance of vision feedback.^384,385^ However, fluorescent probes (e.g., organic dyes,^386^ quantum dots,^387^ metal–organic frameworks,^388,389^ etc.), which usually have poor biocompatibility and biodegradability, are required to label the micro/nanorobotic materials or cells. Because of the intrinsic fluorescence feature, excellent biocompatibility, and biodegradable performance of Spirulina microalgae, microalgae-based magnetic microrobots allow for *in vivo* fluorescent imaging without the use of probes and concern for biosafety (Figure 19C).^249^

Ultrasound imaging, as a conventional clinical imaging technique, mainly has two different modalities, namely, B-mode and Doppler.^390,391^ The former is based on pulse–echo technique while the latter relies on the Doppler effect. The main advantages of US imaging lie in high spatial and temporal resolution, large penetration depth, minimal damage to tissues, and relatively lower setup cost. A magnetically driven microrobot swarm was visualized and tracked in a bovine eyeball via US imaging^125^ as shown in Figure 19D. Sitti’s group used the color Doppler mode of US imaging to track the “hairbots” in *ex vivo* chicken breast.^268^ Recently, Zhang’s group adopted US Doppler for real-time guidance of a swarm of magnetic microrobots for endovascular delivery.^370^

Photoacoustic imaging, first proposed by Alexander Graham Bell^392^ in 1881, is a “light-in, sound-out” approach. A light source (i.e., IR laser) and US transducer are two fundamental elements for a PA imaging setup. Utilization of PA imaging to track microalgae-based magnetic microswimmers for killing pathogenic bacterial was reported.^226^ A more advanced PA imaging technique, multispectral optoacoustic tomography, was adopted for real-time monitoring of the migration of single magnetically driven conical micromotors with the length of 100 μm in phantom as well as *ex vivo* chicken tissue^393^ as show in Figure 19E.

X-ray-CT, PET, and SPECT belong to the category of ionizing radiation-based techniques that employ high-frequency radiation with wavelength ranging 10–100 nm. As a consequence, these techniques endow high penetration depth and spatial resolution, but the harm radiation does to living (human) tissues must be taken into consideration. In comparison with the widely used X-ray CT technique used in clinics, PET and SPECT techniques based on γ-rays have been developed in the last decades. Although the two state-of-the-art imaging techniques exhibit excellent spatial resolution and molecular selectivity, the utilization of PET and SPECT (usually in conjunction with CT imaging) for the localization and tracking of MagRobots is still in its infancy. For both techniques, interested materials or micro/nanorobots are often conjugated with radiotracers (such as ^64^Cu, ^124^I, ^18^F, ^68^Ga, ^99m^Tc, etc.).^374,378,394−397^ SPECT imaging for individual microrobots with diameter as low as 100 μm was reported by Nelson’s group^374^ as shown in Figure 19F. To track the shape transition (e.g., from tubular to planar configuration) of microrobots, they used ^99m^Tc [Tc]-based radioactive compounds to label the magnetically driven thermoresponsive hydrogel-based microrobots. More research is expected to explore the combination between biomedical imaging techniques and locomotive micro/nanorobots, and aimed at targeting individual MagRobots or a swarm of MagRobots to a specific location with high temporal and spatial precision, and executing certain diagnostic or therapeutic tasks in an invasive and visualizable fashion. Because of the restriction of small size, clear observation of a single miniaturized robotic in the nanoscale and microscale using current biomedical imaging techniques is still a big challenge.

### 5.7 Pollution Removal for Environmental Remediation

**Figure 20:**
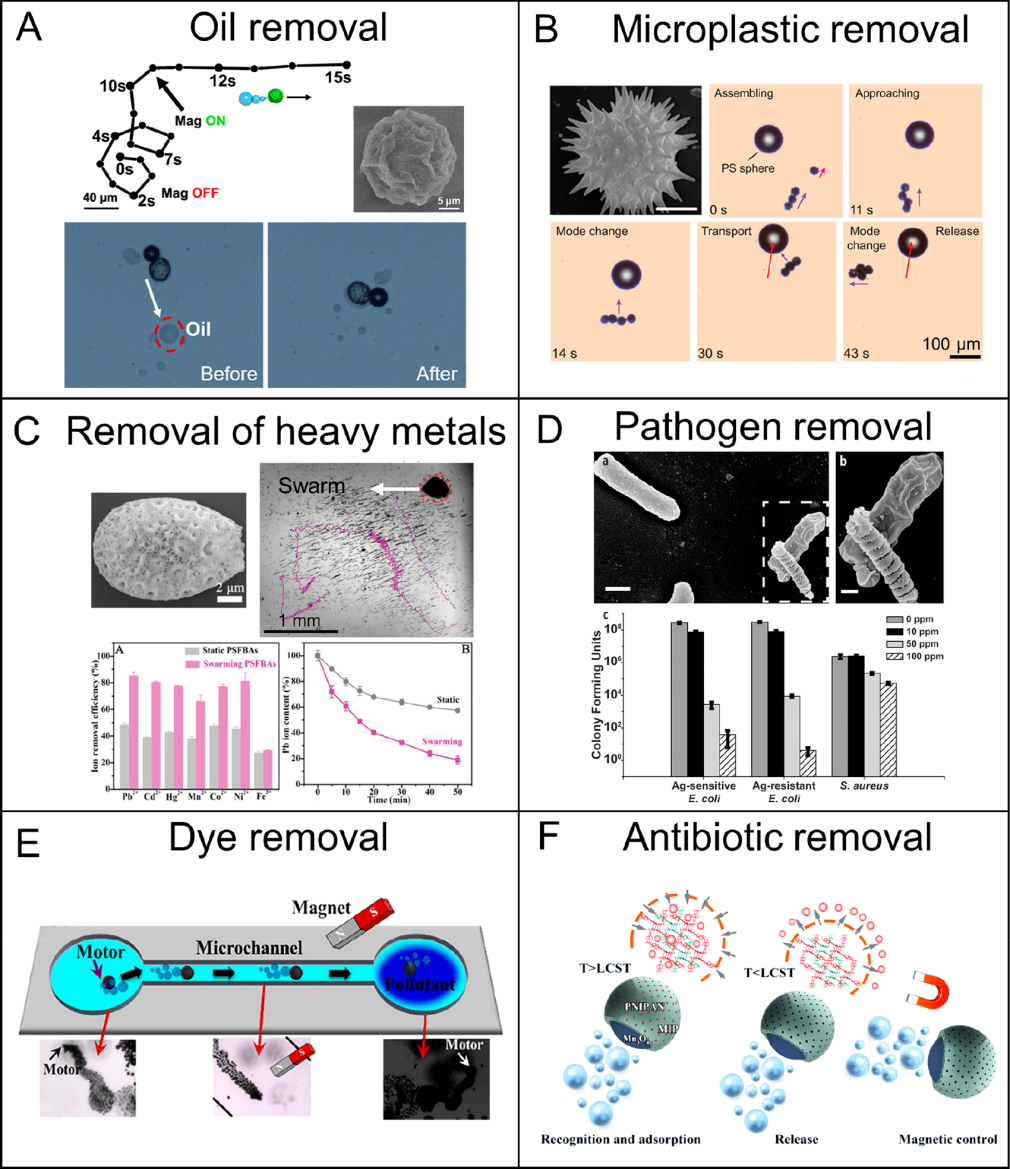
Representative pollutant removal by active MagRobots. (A) Directional motion of walnut-like magnetic micromotor under an external magnetic field and its oil-removal ability. Reproduced with permission from ref (401). Copyright 2019 American Chemical Society. (B) Pollen-based microsubmarines for the removal of microplastics (i.e., PS spheres). Reproduced with permission from ref (402). Copyright 2020 Elsevier Ltd. (C) Higher removal efficiency of heavy metals by dynamically swarming spore@Fe_3_O_4_ biohybrid micromachines compared with that of their static counterparts. Reproduced with permission from ref (292). Copyright 2018 WILEY-VCH Verlag GmbH and Co. KGaA, Weinheim. (D) Pd/Ni/Ag nanocoils for removing microbial pathogens. Reproduced with permission from ref (403). Copyright 2015 WILEY-VCH Verlag GmbH and Co. KGaA, Weinheim. (E) Magneto-catalytic micromotors for the degradation of Methylene Blue (MB) dye. Reproduced with permission from ref (404). Copyright 2020 American Chemical Society. (F) Lotus pollen-templated magnetic micromotors for temperature-sensitive adsorption of erythromycin. Reproduced with permission from ref (290). Copyright 2019 Elsevier B.V.

In addition to the biofriendliness, recoverability of magnetically driven micro- and nanorobots, and the toxin-free nature of magnetic manipulation, MagRobots can also actively swim around waterborne pollutants (e.g., dyes, oil, heavy metals,^292^ microplastics, microbial pathogens, estrogenic,^398,399^ etc.) and remove them by capture (adsorption/absorption) or degradation. As such, small-scale MagRobots constitute a technology with great potential for water remediation. In the future, sophisticated magnetic manipulation systems could be used to externally guide MagRobots to pollution sites (i.e., canalizations, industrial reactors, tanks, pools) in a contactless fashion. Additionally, magnetic fields can be used to accelerate reaction kinetics or recognition efficiency due to the robust dynamic intermixing (i.e., magnetic stirring function) and to retrieve the nano/microrobots once the cleaning procedure has been finalized.^400^ Eventually, the cleaning agents can be reused or recycled if their constituent components have remained unaltered. The treatment of six representative pollutants using miniaturized magnetic motors is summarized in Figure 20.

The autonomous movement of a walnut-like microrobot composed of polycaprolactone, Fe_3_O_4_ nanoparticles, and catalase in H_2_O_2_-included solution is ascribed to the oxygen bubbles from the enzyme-catalytic degradation of H_2_O_2_, exhibiting a spiral trajectory.^401^ The direction of the microswimmers could be controlled using external magnetic fields. Because of the hydrophobic nature, the motile walnut-like micromotor was capable of collecting spilled oil (Figure 20A). Because of the incorporation of Fe_3_O_4_ component, the recycling of the micromotor was realized by using a magnetic field.^401^ A magnetic hollow microsubmarine, using natural sunflower pollen grains as a template, was reported to remove leaked oil and microplastics pollutants simultaneously (Figure 20B).^402^ High removal efficiency of heavy metal ions was found in porous biohybrid microrobots consisting of fungi spore and Fe_3_O_4_ nanoparticles. The collective behaviors of the microrobots and magnetically steered agitation could further enhance the pollutant adsorption ability compared with static microrobots (Figure 20C).^292^ The excellent antibacterial ability of Pd/Ni/Ag nanocoils and high magnetic maneuverability at low magnetic strength (8 mT; 10 Hz) allows for precise locomotion of nanorobots toward the target location of bacterial infection to efficiently fight against the drug-resistant bacteria (Figure 20D).^403^ The dual actuation of micromotors prepared from carbon soot by using a magnetic field and oxygen microbubbles facilitated efficient on-the-fly degradation of MB dye pollution^404^ (Figure 20E). In addition, the use of functional magnetic micromotors for the absorption or removal of antibiotics, such as erythromycin (Figure 20F)^290^ and doxycycline^405^ in contaminated water, has also been investigated.

### 5.8 Sensing and Biosensing

**Figure 21:**
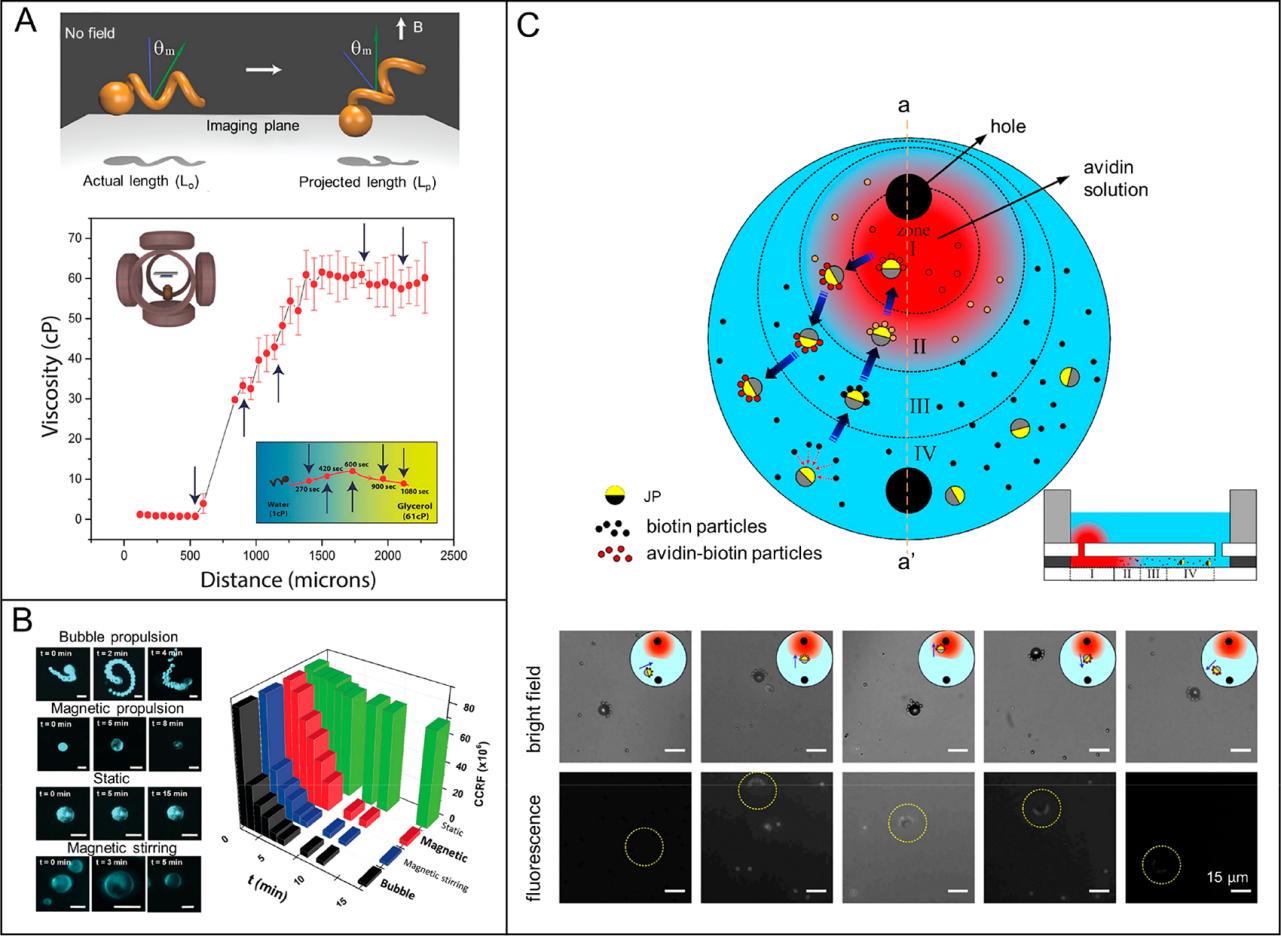
(A) Helical nanorobots as mobile viscometers. Reproduced with permission from ref (158). Copyright 2018 WILEY-VCH Verlag GmbH and Co. KGaA, Weinheim. (B) Graphene Quantum Dots MagRobots for the detection of endotoxin from *E. coli*. Reproduced with permission from ref (408). Copyright 2017 WILEY-VCH Verlag GmbH and Co. KGaA, Weinheim. (C) Janus micromotors deliver biotin-functionalized cargos for avidin sensing within a microfluidic device. Reproduced with permission from ref (409). Copyright 2020 American Chemical Society.

According to sensing mechanisms, there are three main purposes of using magnetically driven micro/nanomotors for sensing and biosensing. First, because the motion behaviors (e.g., velocity, wobbling angle) of MagRobots is related to an applied external magnetic field as well as properties (e.g., temperature, pH, viscosity, ionic strength) of the solution, the detection of these movement parameters of MagRobots provides a novel approach to probing the local microenvironment in a heterogeneous medium.^406^ For instance, a helical nanomotor was developed as a mobile viscometer capable of monitoring in real-time the surrounding viscosity in homogeneous or heterogeneous media. A mathematical model was developed that establishes a relation between viscosity and the precession angle of the swimmer. High temporal and spatial precision of the viscometer was confirmed by gradually measuring the viscosity of deionized water from the hot state (70 °C) to its cool-down state (30 °C) and mapping the local viscosity from a reference fluid (e.g., deionized water) to another fluid (e.g., glycerol–water 4:1 v/v) in a microfluidic chamber under the application of homogeneous rotating magnetic fields (Figure 21A).^158^ Second, externally maneuvered MagRobots can act as signal amplifiers and, therefore, provide enhanced detection sensitivity and efficiency for identifying the signals (e.g., fluorescence) triggered by target molecules due to the active stirring and vigorous mass transfer in the solution.^407^ Janus micromotors, which contain phenylboronic acid-modified graphene quantum dots, iron oxide nanoparticles, and Pt nanoparticles, were used to detect the bacterial endotoxin in contaminated water. The reaction between graphene quantum dots and the targeted endotoxin results in the fluorescence quenching of the dots while phenylboronic acid tags serve as specific recognition receptors of the endotoxin. Compared with that in the static conditions, the micromotors actuated by external magnetic fields or those autonomously propelled by oxygen bubbles displayed faster fluorescence quenching than those that remained static due to elevated fluid intermixing (Figure 21B).^408^ Similarly, mobile magnetic spore@Fe_3_O_4_@CDs microrobots can remotely detect *C. diff* toxins with much more obvious fluorenes quenching in a noninvasive way through the targeting combination of *C. diff* toxins and CDs (carbon quantum dots) in comparison with nonactuated microrobots.^296^ Third, MagRobots can function as a navigator, precisely guiding payloads (especially biomolecules for the diagnostic purpose) to a user-defined site for chemical/biological interactions or other purposes in an untethered way. Janus magnetic microrobots were capable of loading biotin-functionalized commercially purchased microbeads and transporting them to a specific region under the steering of a uniform electric field and rotating magnet. The dynamic binding between the surface-immobilized probe (i.e., biotin) and the target analyte (i.e., avidin) provides a label-free method for biosensing. The experimental detection limit in a single microfluidic chamber can be as low as 2 μg/mL (Figure 21C).^409^

## 6 Conclusion and Future Perspectives

The last decades have witnessed great advances and breakthroughs in MagRobots, including innovative manufacturing approaches, reconfigurable and programmable navigation techniques, advanced theoretical models, impressive proofs of concept, and clinically oriented application trials. This review introduces basic knowledge of magnetic fields and magnetic materials, offers the experimental setups of magnetic manipulation systems and various field configurations, and proposes the strategies to generate nonreciprocal movement. The movement mechanisms of flagella-inspired helical motion, undulatory motion, and boundary-assisted motion also are presented. Fabrication techniques of (quasi-)spherical, helical, flexible, wire-like, and biohybrid MagRobots are summarized, followed by various state-of-the-art applications in the field of biomedicine and environment.

The considerable application potential of micro/nanorobots in the biomedical area, such as targeted drug/gene delivery, localized bioanalysis, cell sorting, microsurgery, biopsy, detoxification, biofilm removal, and biosensing becomes a driving force that attracts an increasing number of scientists to join in this emerging research field.^144,410^

In addition, before implementing MagRobots in real applications, the following aspects should be taken into consideration: (i) MagRobots’ materials should meet the standards of practical biomedical and environmental applications, such as biocompatibility and biodegradability, and bring economic and social benefit. For instance, expensive materials and fabrication apparatus or complicated preparation procedures limit the mass production of synthetic microstructures. This is a challenge that researchers face today and should be solved in the future. (ii) To enhance the work efficiency of MagRobots in complex environments, swarms or collective behavior of synthetic MagRobots can be regulated to cooperatively and efficiently execute complex biological or environmental missions that would be insurmountable for a single MagRobot. Moreover, reconfigurability provides another strategy for MagRobots to adapt to variational biological surroundings. For instance, the intriguing collective behavior from the self-assembly of nanoparticles could present a reversible pattern transformation (i.e., reconfigurability) under the steering of an external field, enhancing MagRobots’ tasking capabilities and high environmental adaptability. Finally, great endeavors have been made to navigate these untethered microrobots in various complex body fluids such as blood, gastric juice, urine, cerebrospinal fluid,^217^ and intracellular medium. However, given the complexity of biological fluids, the relation between movement behaviors of MagRobots and environment parameters (e.g., the components, temperature, viscosity, boundaries, the flow speed of the biological fluids, etc.) are expected to be theoretically and experimentally established in order to obtain better control of MagRobots. (iii) Precise maneuvering of MagRobots on-body and in real-time is very important and their monitoring is essential. This is a challenge confronted by micro/nanorobots researchers. Clinical imaging systems in current use, such as MRI as discussed in Section 5.6, can help in terms of visualization and as an actuation source. However, there is still room to improve MagRobots’ programmability in terms of orientation, locomotion, and even morphology. In this way, if MagRobots can be controlled and altered according to actual conditions or occasions such as the patient’s health status and physiology, then MagRobots will be able to perform precise and personalized therapy.

**Figure 22:**
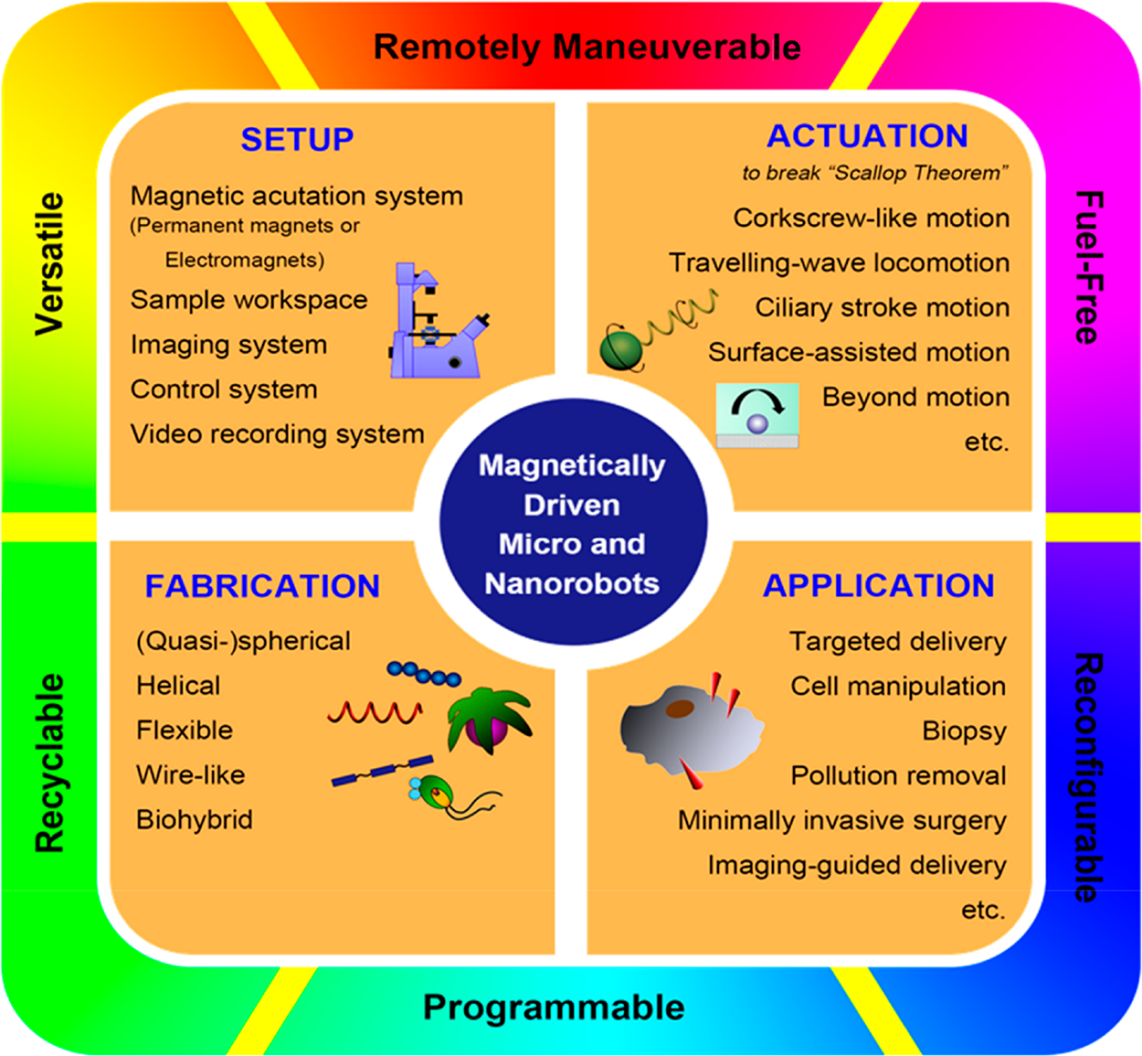
Diagrammatic summary of this review including (but not limited to) experimental setups, actuation mechanisms, fabrication approaches for various MagRobots, and applications, and the advantages of MagRobots.

In summary, a good understanding of the mechanism of magnetically driven micro/nanorobots and corresponding impact factors (e.g., geometrical shape, field configuration, fluids properties, and boundary) is a precondition for the conceptualization, functionalization, and automation of MagRobots. High spatial maneuverability, fast reconfigurability, and precise programmability are the ultimate research goals of small-scale robots (see Figure 22). Although there is a long way to go to translate robust minimized robots from bench to bedside, considerable advances are bringing fantasy closer to reality.
